# Elucidating Ion Transport Phenomena in Sulfide/Polymer Composite Electrolytes for Practical Solid-State Batteries

**DOI:** 10.1007/s40820-023-01139-w

**Published:** 2023-07-13

**Authors:** Kyeong-Seok Oh, Ji Eun Lee, Yong-Hyeok Lee, Yi-Su Jeong, Imanuel Kristanto, Hong-Seok Min, Sang-Mo Kim, Young Jun Hong, Sang Kyu Kwak, Sang-Young Lee

**Affiliations:** 1 Department of Chemical and Biomolecular Engineering, Yonsei University, 50 Yonsei-ro, Seodaemun-gu, Seoul, 03722 Republic of Korea; 2 Department of Chemical and Biological Engineering, Korea University, 145 Anam-ro, Seongbuk-gu, Seoul, 02841 Republic of Korea; 3 School of Energy and Chemical Engineering, Ulsan National Institute of Science and Technology (UNIST), 50 UNIST-gil, Eonyang-eup, Ulju-gun, Ulsan, 44919 Republic of Korea; 4 Hyundai Motor Company, 150, Hyundaiyeonguso-ro, Namyang-eup, Hwaseong-si, Gyeonggi-do 18280 Republic of Korea

**Keywords:** Solid-state batteries, Composite solid-state electrolytes, Ion transport phenomena, Bi-percolating ion channels, Interfacial resistance

## Abstract

Despite the enormous interest in inorganic/polymer composite solid-state electrolytes (CSEs) for solid-state batteries (SSBs), the underlying ion transport phenomena in CSEs have not yet been elucidated. Here, we address this issue by formulating a mechanistic understanding of bi-percolating ion channels formation and ion conduction across inorganic-polymer electrolyte interfaces in CSEs. A model CSE is composed of argyrodite-type Li_6_PS_5_Cl (LPSCl) and gel polymer electrolyte (GPE, including Li^+^-glyme complex as an ion-conducting medium). The percolation threshold of the LPSCl phase in the CSE strongly depends on the elasticity of the GPE phase. Additionally, manipulating the solvation/desolvation behavior of the Li^+^-glyme complex in the GPE facilitates ion conduction across the LPSCl-GPE interface. The resulting scalable CSE (area = 8 × 6 (cm × cm), thickness ~ 40 μm) can be assembled with a high-mass-loading LiNi_0.7_Co_0.15_Mn_0.15_O_2_ cathode (areal-mass-loading = 39 mg cm^–2^) and a graphite anode (negative (N)/positive (P) capacity ratio = 1.1) in order to fabricate an SSB full cell with bi-cell configuration. Under this constrained cell condition, the SSB full cell exhibits high volumetric energy density (480 Wh L_cell_^−1^) and stable cyclability at 25 °C, far exceeding the values reported by previous CSE-based SSBs.

**Supplementary Information:**

The online version contains supplementary material available at 10.1007/s40820-023-01139-w.

## Highlights

- Mechanistic understanding of ion transport phenomena in composite solid-state electrolytes (CSEs) for practical solid-state batteries is conducted.
- Percolation threshold formation of the inorganic (LPSCl) phase in the CSEs depends on elasticity of the gel polymer electrolyte (GPE) phase.
- Manipulating the solvation/desolvation behavior of the GPE phase facilitates ion conduction across the LPSCl-GPE interfaces.

**Supplementary Information:**

The online version contains supplementary material available at 10.1007/s40820-023-01139-w.

## Graphical abstract

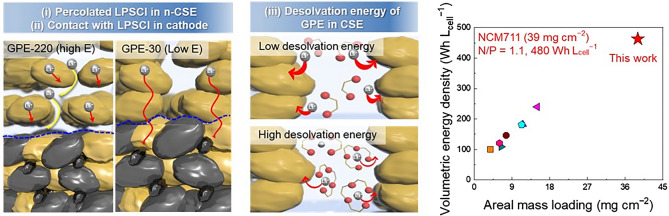

## Introduction

The ever-increasing demand for high-energy–density/high-safety batteries has inspired the relentless pursuit of solid-state batteries (SSBs) as a promising alternative to current state-of-the-art lithium (Li)-ion batteries [1, 2]. Many previous studies on SSBs have employed inorganic electrolytes based on sulfides/oxides [3, 4] as a solid-state ion conductor. Unfortunately, the practical applicability of inorganic electrolyte-containing SSBs has been hampered by grain-boundary/interfacial resistances, limited chemical stability, hygroscopicity, mechanical rigidity, and complicated cell fabrication [5–7]. Alongside inorganic electrolytes, polymer electrolytes have also been considered as a potential candidate due to their mechanical flexibility, conformability, and intimate contact with electrodes. However, their low ionic conductivity, narrow electrochemical stability window, and insufficient mechanical strength have hindered their applications to SSBs [8–10].

As a facile and practical approach to address the challenges posed by inorganic and polymer electrolytes, composite solid-state electrolytes (CSEs), which are designed to couple the complementary features of individual inorganic and polymer electrolytes, have also been investigated [11–14]. However, most previously reported CSEs [15–19] have mainly focused on their ionic conductivity and electrochemical stability with electrode materials, with little attention to the underlying ion transport phenomena. One common misconception regarding the CSEs is that their ionic conductivity shows a linear proportionality with composition ratios. Considering that the ion conduction mechanism of inorganic electrolytes is completely different from that of polymer electrolytes [20, 21], this linear relationship between ionic conductivity and composition ratios is not universally accepted. For example, given that inorganic electrolyte particles are dispersed in a polymer electrolyte matrix without being interconnected, the ionic conductivity of the resulting CSE is predominantly determined by the polymer electrolyte matrix rather than its composition ratios. This result underscores the co-continuous percolation of inorganic and polymer electrolyte phases in the CSEs. Another crucial issue that must be urgently resolved is alleviating ionic resistances across inorganic-polymer electrolyte interfaces [22].

Here, we elucidate ion transport phenomena in the CSEs, focusing particularly on bi-percolating ion channel formation and ion conduction across inorganic-polymer electrolyte interfaces. Argyrodite-type Li_6_PS_5_Cl (LPSCl) and gel polymer electrolytes (GPEs) (consisting of a Li^+^-glyme complex as an ion-conducting medium and a crosslinked ethoxylated trimethylolpropane triacrylate (ETPTA) polymer as a mechanical skeleton) are chosen as model inorganic and polymer electrolyte systems, respectively. The effect of the LPSCl/GPE composition ratio on ionic conductivity of the CSE is investigated and compared with a control CSE (comprising alumina (Al_2_O_3_) nanoparticles (chosen as an ionically inert inorganic counterpart to LPSCl) and GPE). The dependence of the percolation threshold formation of the LPSCl phase on the elasticity of the GPE phase is elucidated as a function of fabrication pressure applied during the CSE manufacturing process. To the best of our knowledge, this elasticity effect of polymer electrolytes on the phase morphology has not been considered in CSE design elsewhere. Additionally, ion transport across the LPSCl-GPE interface is facilitated by manipulating the solvation/desolvation behavior of Li^+^-glyme complexes in the GPEs. This understanding of ion transport phenomena in the CSE can be used as a versatile platform for advanced CSE design based on various sulfide/polymer electrolyte mixtures.

The resulting CSE which has optimal material chemistry and composition ratios, is combined with an aramid nonwoven porous substrate (acting as a mechanical scaffold) to achieve manufacturing scalability and mechanical flexibility. The nonwoven-embedded CSE (area = 8 × 6 (cm × cm), thickness ~ 40 μm) is assembled using a high-mass-loading LiNi_0.7_Co_0.15_Mn_0.15_O_2_ cathode (39 mg cm^–2^, corresponding to an areal capacity of 3.5 mAh cm^−2^) and a graphite anode (negative (N)/positive (P) capacity ratio = 1.1) in order to fabricate a SSB full cell with bi-cell configuration. Under this constrained cell condition, the SSB full cell exhibits high energy density (480 Wh L_cell_^−1^) and stable cyclability at 25 °C. This result demonstrates the viability of the rationally designed CSE in enabling a practical SSB full cell.

## Experimental Section

### Preparation of the CSE

Argyrodite-type Li_6_PS_5_Cl (LPSCl, average particle size ~ 3 µm) was prepared by ball-milling (Pulverisette 7 PL, Fritsch GmbH) and subsequent heat treatment under Ar atmosphere. A stoichiometric mixture of Li_2_S (99.9%, Alfa-Aesar), P_2_S_5_ (99%, Sigma-Aldrich), and LiCl (99.99%, Sigma-Aldrich) was ball-milled at 600 rpm for 10 h. The resulting powders were annealed at 550 °C for 5 h. Meanwhile, the Li^+^-glyme complexes were prepared by mixing anhydrous glymes (Sigma-Aldrich) (dimethoxyethane (G1), diethylenegylcoldimethylether (G2), and triethylene glycol dimethyl ether (G3)) with lithium bis(fluorosulfonyl)imide) (LiFSI) (LG Energy Solution) at an equimolar ratio. The composite solid-state electrolytes (CSEs) were fabricated by mixing the LPSCl with the GPE precursor (Li^+^-glyme complexes = 1/1 (mol mol^−1^), and UV-curable ethoxylated trimethylolpropane triacrylate (ETPTA) monomer (incorporating 5 wt.% 2-hydroxy-2-methylpropiophenone (HMPP) as a photoinitiator)) at varied composition ratios. The obtained mixtures of LPSCl/GPE precursor were subjected to ultrasonication (for 2 h) followed by ball milling (for 0.5 h) to achieve a good dispersion state. Subsequently, the mixtures were exposed to UV irradiation for less than 30 s to crosslink ETPTA polymer skeleton [23–25], in which the UV irradiation was performed using an Hg UV-lamp (Lichtzen) with an irradiation peak intensity of approximately 2000 mW cm^−2^. An optimal composition ratio of the CSE was set to LPSCl/GPE = 70/30 (v/v) at an environmental pressure of 74 MPa. The GPE-30 and GPE-220 were composed of LiG3/ETPTA = 96.5/3.5 (w/w) and 85/15 (w/w), respectively. Meanwhile, the CSE was combined with an aramid nonwoven (thickness ~ 18 µm, porosity ~ 86%, average pore size ~ 110 µm, Dupont Inc.) to produce a nonwoven-embedded CSE (n-CSE, thickness ~ 40 µm), in which a composition ratio of the GPE mixture in the CSE was LiGX (X = 1, 2, and 3)/ETPTA monomer = 96.5/3.5 (w/w).

The CSE slurry (i.e., prior to UV curing) was impregnated into the aramid nonwoven porous substrate and then solidified after exposure to UV irradiation for less than 1 min on each side of the CSE layer, finally producing the n-CSE membrane by the mechanical pressing at 370 MPa.

### Physicochemical/Electrochemical Characterization of the CSE

The XRD patterns were recorded a MiniFlex 600 diffractometer (Rigaku Corp.) operated at 40 kV and 40 mA with Cu Kα radiation of 1.5406 Å. The Young’s modulus (E) of the GPEs was investigated by Nanoindentation (TS1, Hysitron). The morphologies of the CSEs and their components were characterized using field-emission scanning electron microscopy (FE-SEM) (S4800, Hitachi) equipped with energy-dispersive X-ray spectroscopy (EDS). The pore size distribution of the nonwoven substrate and n-CSE was investigated by using mercury intrusion porosimetry (Auto Pore IV 9520 (Micromeritics)). The mechanical flexibility of the n-CSE was quantitatively measured using a universal testing machine (DA-01, Petrol LAB). The ionic conductivities were measured with Li^+^ blocking symmetric cells based on an electrochemical impedance spectroscopy (EIS) analysis at a frequency range from 10^−2^ to 10^6^ Hz and an applied amplitude of 10 mV. The electrochemical performance was investigated using a pellet-type cell and a potentiostat (VSP classic, (Bio-Logic)). The Li^+^ transference number (*t*_Li+_) was evaluated using a potentiostatic polarization method. The DC polarization through a Li^+^ non-blocking symmetric cell and its sequential EIS before and after the polarization was analyzed to determine the Li^+^ transference number [26]:

1 $$ t_{Li + } = \frac{{I_{s} \left( {\Delta V - I_{o} R_{o} } \right)}}{{I_{o} \left( {\Delta V - I_{s} R_{s} } \right)}} $$

where Δ*V* is applied potential, *I*_o_ and *R*_o_ are the initial current and resistance, and *I*_s_ and *R*_s_ are the steady-state current and resistance after the polarization, respectively. To track Li^+^ pathways, ^6^Li^+^ non-blocking symmetric cells of ^6^Li|CSEs|^6^Li were assembled. ^6^Li foils were attached to both sides of the CSE pellets. The solid-state NMR experiments were carried out using an Varian VNMRS 600 MHz FT-NMR spectrometer equipped with 1.6 mm HXY fast MAS probe and spinning at 35 kHz. ^7^Li chemical shifts were referenced to a 1.0 M aqueous LiCl solution at 0.0 ppm as an external standard. The Li plating/stripping cyclability of Li (100 µm)||Li (100 µm) symmetric cells was investigated under a current density of 1 mA cm^−2^ and an areal capacity of 1 mAh cm^−2^ at 25 °C.

*Fabrication and structural characterization of the SSB full cells with the CSEs*: The electrode slurries were prepared by mixing cathode active materials (LiNbO_3_ (1.4 wt.%)-coated LiNi_0.7_Co_0.15_Mn_0.15_O_2_ (NCM711)) [27] or anode active materials (graphite (Gr)), LPSCl, polybutadiene rubber as a binder, and super C65 (only for the NCM711 cathode) as a conductive additive in anhydrous xylene. The composition ratios (electrode active material/LPSCl/binder/super C65) of the electrodes were 74.5/21.5/2.0/2.0 (w/w/w/w)) for the NCM711 cathode and 75.0/22.0/3.0/0.0 (w/w/w/w)) for the Gr anode, respectively. The electrode slurries were casted on current collectors (Al (for the NCM711 cathode) and Ni foils (for the Gr anode)) using a doctor blade method, followed by heat treatment at 120 °C under vacuum [28]. The N/P ratio (the areal capacity ratio between the negative (N) and positive (P) electrodes) was fixed as 1.1. Meanwhile, the self-standing n-CSE membranes (thickness ~ 40 μm) were fabricated by impregnating the above-prepared CSE precursors (composed of the LPSCl/GPE precursors) into aramid nonwoven porous substrates by pressing at 370 MPa and then solidifying after exposure to UV irradiation. The resulting n-CSE membranes (13 mm in diameter) were assembled with the preformed NCM711 cathodes (10 mm in diameter) and graphite anodes (12 mm in diameter). Subsequently, the NCM711 cathode/n-CSE-membrane/graphite anode assembly was subjected to pressing at 74 MPa at 25 °C to produce SSB full cell. To fabricate the SSB full cell with the bi-cell configuration, two Gr anodes were assembled with a double-sided NCM cathode (areal capacity = 3.5 mAh cm^−2^). All cell assembly processes were performed in a polyaryletheretherketone (PEEK) mold (1.33 cm^2^) with two Ti metal rods. The internal structure of the SSB full cell was characterized by X-ray CT (Xradia 520 Versa three-dimensional (3D) X-ray microscope, Zeiss), in which the SSB full cell was sealed with a pouch packaging for this structural analysis.

### Electrochemical Characterization of the SSB Full Cells

The cycling performance of the SSB full cells was examined using a cycle tester (PNE Solution Co., Ltd, Korea) at charge/discharge current density of 0.05 C (= 0.088 mA cm^−2^)/0.05 C under a voltage range of 2.5–4.3 V (vs. Li/Li^+^). For the rate capability test, the cells were cycled between 2.5 and 4.3 V at discharging current rates ranging from 0.05 C to 2.0 C under a constant charging rate of 0.05 C. The electrochemical performance of the pellet-type SSB full cells was characterized under a fixed pressure of 74 MPa. The impedance of the SSB full cell was measured by EIS at a frequency range from 10^−2^ to 10^6^ Hz and an applied amplitude of 10 mV. All the processes were carried out in an Ar-filled glove box.

### MD Simulations

**Fig. 1:**
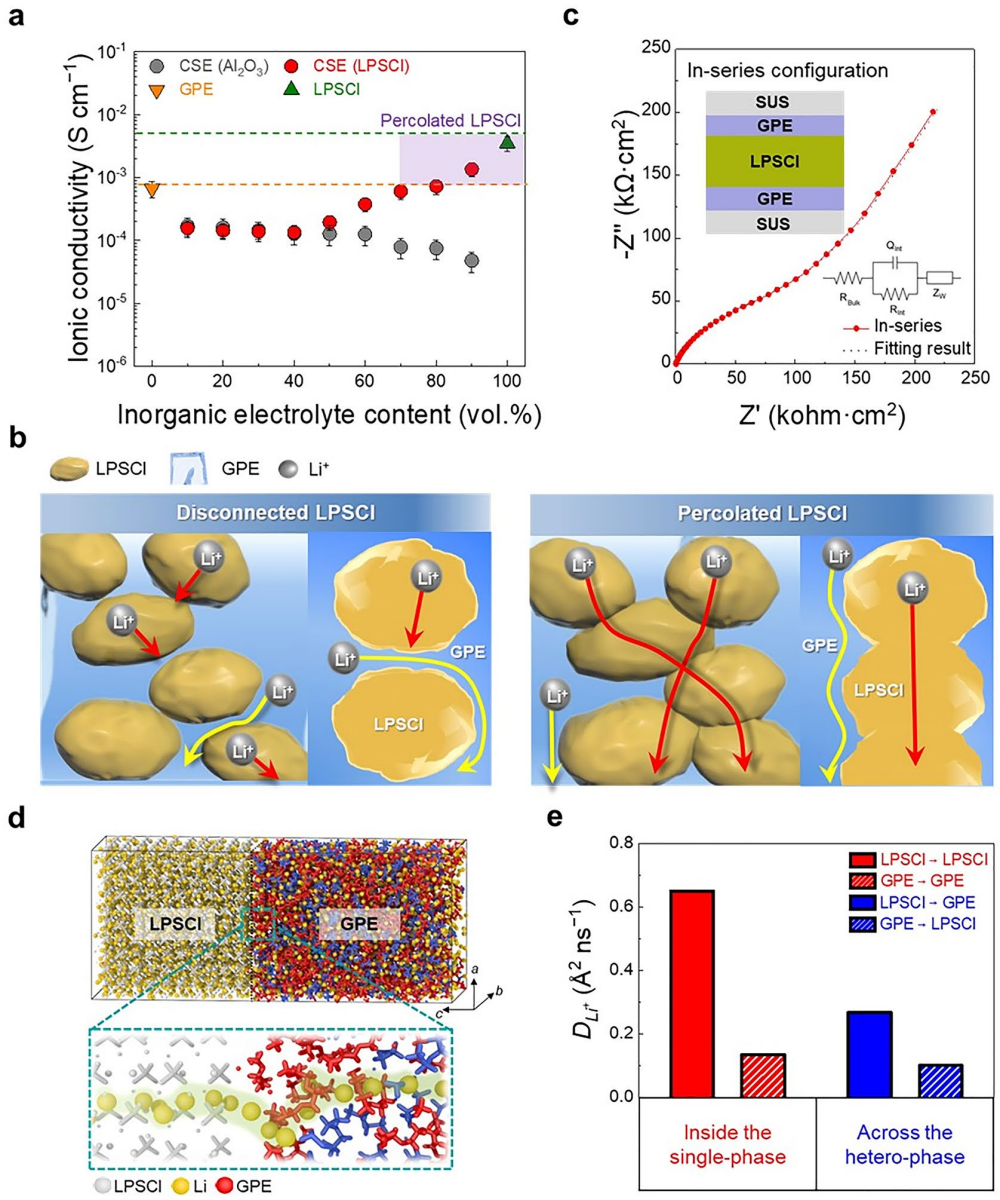
Elucidating ion transport phenomena of the CSEs. **a** Ionic conductivity of the CSE as a function of LPSCl (and Al_2_O_3_) content under a constant environmental pressure of 74 MPa and a temperature of 25 °C. The ionic conductivities of the samples were repeatedly measured three times to ensure the data reliability. **b** Schematic illustration depicting the influence of phase morphology on the ion conduction of the CSE. **c** Nyquist plot of a tri-layered GPE/LPSCl/GPE model electrolyte. Insets show a schematic depicting the in-series configuration of the model electrolyte and corresponding equivalent electric circuit model. **d** Model systems used to simulate Li^+^ conduction across the LPSCl-GPE interface (considered as a hetero-phase electrolyte) in the CSE. **e** Diffusion coefficients of Li^+^ (*D*_Li+_) inside the single-phase electrolytes (LPSCl and GPE, respectively) and the hetero-phase electrolyte in the CSE. Note that the Figure legends of LPSCl → LPSCl and GPE → GPE indicate Li^+^ conduction inside the pristine (i.e., single-phase) electrolytes. The Figure legends of LPSCl → GPE and GPE → LPSCl indicate Li^+^ conduction across the two different (i.e., hetero-phase) electrolytes

We performed molecular dynamics (MD) simulation to investigate the Li^+^ conduction behavior in LPSCl, GPE, and LPSCl-GPE interfacial systems and to observe the change of Li^+^ solvation free energy in the GPE systems with different chain lengths. The LPSCl system was modeled with 5 × 5 supercells of LPSCl (100) surface (i.e., lattice parameters: *a* = *b* = 41.12Å, *c* = 51.42Å, α = β = γ = 90˚) which was found to be the most stable surface (i.e., DFT calculated surface energy of LPSCl (100), (010), and (111) are 0.060, 0.286, and 2.059 eV nm^−2^, respectively). We assumed dynamically rigid structure to describe the LPSCl crystal structure. The GPE systems were modeled by packing 300 molecules of LiGX (X = 1, 2, and 3) and ETPTA molecules with the same composition as used in the experimental setup (i.e., equimolar mixtures of LiGX with 15 and 3.5 wt.% ETPTA polymer in the GPEs, respectively, as shown in Figs. 1e and 4g). Furthermore, the LPSCl-GPE interface systems were constructed by assembling the separately built LPSCl and GPE model systems with the same *a* and *b* lattice. The calculations were performed with NVT ensemble in electrolyte systems at 25 °C with 1 fs timestep for 5 ns [29]. The mean square displacement (MSD) analysis was conducted using the last 500 ps trajectory, and the diffusion coefficients of Li^+^ (*D*_Li+_) were calculated based on the slope of the linear fitted MSD plot through the Einstein equation [30]. To investigate Li^+^ solvation free energy with different chain lengths of glyme, LiGX (X = 1, 2, and 3) systems were constructed with 150 LiFSI molecules and 150 glyme molecules to represent the equimolar ratio. All systems were subjected to 500 ps NPT simulation to equilibrate the systems. Subsequently, the solvation free energies were calculated through Acceptance Ratio algorithm [31] with NVT ensemble at 25 °C with 1 fs timestep. The coupling parameter was divided into 12 windows, for each run 250,000 equilibration steps and 500,000 production steps were carried out. The MD simulation was performed using the Forcite module in the Materials Studio 2019 [32]_,_ with COMPASSII force field [33] to describe bonding and non-bonding interactions in the system. The temperature control was done using Nose–Hoover Langevin (NHL) thermostat [34] and pressure control was done using Berendsen barostat [35]. The summations for electrostatic and van der Waals (vdW) interactions were done using Ewald and atom-based method (i.e., cut-off radius = 15.5 Å), respectively. The visualization was performed using 3D visualization Open Visualization Tool (OVITO) [36].

## Results and Discussion

### Elucidating Ion Transport Phenomena of CSEs

As an initial step in the preparation of CSEs, we investigated the chemical stability of their components. An initially transparent triglyme (G3) solvent turned yellow upon contact with LPSCl (Fig. S1a). By comparison, an equimolar mixture of lithium bis(fluorosulfonyl)imide (LiFSI)/G3 (= 1/1 (mol/mol), denoted as LiG3) remained inert, which can be explained by the hard and soft acids and bases (HSAB) theory [37, 38]. Similar to the LiG3, a LiG3/ETPTA monomer mixture (= 85/15 (w/w)) was not affected by the LPSCl. This result was further verified by X-ray diffraction (XRD) analysis. After being kept in the LiG3/ETPTA monomer mixture for seven days, the LPSCl remained stable and maintained its characteristic XRD peaks (Fig. S1b). Subsequently, the LPSCl/LiG3/ETPTA monomer mixture was exposed to UV irradiation to crosslink the ETPTA monomer, which led to the formation of the GPE (LiG3/crosslinked ETPTA polymer skeleton) in the presence of LPSCl. After being compressed using an isostatic pressing method at a constant environmental pressure of 74 MPa, a self-standing CSE was obtained (Fig. S2). 

The ionic conductivity (σ) of the CSE was investigated as a function of LPSCl content under a constant environmental pressure of 74 MPa and a temperature of 25 °C (Fig. 1a). The ionic conductivities of the individual GPE and LPSCl were 0.67 and 2.43 mS cm^−1^, respectively. From this result, we predicted that the ionic conductivity of the CSEs would increase with LPSCl content. However, contrary to our expectation, adding 10 vol.% LPSCl into the GPE decreased ionic conductivity (0.67 (GPE) to 0.36 mS cm^−1^ (10 vol.% LPSCl, the obtained CSE and its stepwise fabrication procedure were shown in Fig. S2a) and remained almost unchanged up to 60 vol.% LPSCl. At volumes above 70 vol.% LPSCl, however, the ionic conductivity of the CSE tended to increase with the LPSCl content. This result indicates that the LPSCl phase can form percolating ion channels, thereby contributing to the ionic conductivity of the CSE. To better understand this dependence of the ionic conductivity on the LPSCl content, a control sample (consisting of ionically inert Al_2_O_3_ particles (average particle size ~ 3 μm) and GPE) was prepared and its ionic conductivity was examined as a function of Al_2_O_3_ content. Up to 40 vol.% Al_2_O_3_, the ionic conductivity of the control sample was similar to that of the CSE. However, as the Al_2_O_3_ content was further increased, the ionic conductivity of the control sample continued to decrease, in contrast to the result of the LPSCl-containing CSE. This result shows that the ionic conductivity of the control sample was mainly influenced by the GPE matrix, while the Al_2_O_3_ particles acted as an ionically inert filler. This influence of the phase morphology on the ionic conductivity of the CSE is schematically illustrated in Fig. 1b. From the comparison of the LPSCl with Al_2_O_3_, we found that at the low LPSCl contents, there was no significant difference in the ionic conductivity between the ionically conductive LPSCl and ionically inert Al_2_O_3_. In comparison, at the relatively higher LPSCl contents, the contribution of the LPSCl to the ionic conductivity became more pronounced. This result demonstrates the importance of the LPSCl content in the formation of the percolated LPSCl phase in the CSE. Simultaneously, the difference between the CSE and control sample was further highlighted at a composition ratio of LPSCl (or Al_2_O_3_)/GPE = 70/30 (v/v). The ionic conductivity of the CSE increased with pressure, plateauing at 74 MPa, whereas the control sample showed no change (Fig. S3). This result exhibits that the applied pressure, in addition to the LPSCl content mentioned above, plays a significant role in forming the percolated LPSCl phase in the CSE.

In addition to the percolating ion channel formation described above, we need to elucidate ion conduction behavior across the LPSCl-GPE interface, which is another crucial factor affecting the ionic conductivity of the CSE. As a model study, we measured the interfacial resistance between the individual LPSCl and GPE layers (Fig. 1c), in which a self-standing LPSCl layer (thickness ~ 600 μm) was sandwiched between two GPE layers (thickness ~ 300 μm) forming a tri-layer of GPE/LPSCl/GPE in symmetric cells with blocking electrodes [20, 39]. Electrochemical impedance spectroscopy (EIS) analysis of the symmetric cell showed an ionic resistance of 83.6 kΩ cm^2^ at the LPSCl-GPE interface (Table S1), which was three orders of magnitude larger than those of the individual LPSCl (24.7 Ω cm^2^) and GPE (44.7 Ω cm^2^) layers (Fig. S4). This result shows that ion conduction across the LPSCl-GPE interface is relatively sluggish compared with ion transport via the individual LPSCl and GPE, which is attributable to the difference in the ion conduction mechanism (i.e., Li^+^ diffusion through interstitial sites of the LPSCl [3] and migration of Li^+^-glyme complexes in the GPE [38], respectively).

Ion conduction across the LPSCl-GPE interface in the model system was theoretically investigated and compared with those of the individual LPSCl and GPE using molecular dynamic (MD) simulation (details are described in the Experimental Section and Fig. S5). Three different electrolyte systems were proposed to represent the constituent phases of the CSE: two single-phase electrolyte systems include the pristine LPSCl or GPE, respectively (Fig. S6a), while the LPSCl-GPE interface was considered to be a hetero-phase electrolyte (Fig. 1d). The mean square displacement (MSD) and Li^+^ diffusion coefficient (*D*_Li+_) in these three different electrolyte systems were analyzed to compare their Li^+^ conduction behavior. The MSD and Li^+^ diffusion coefficient of the hetero-phase electrolyte accounted for ion conduction behavior across the LPSCl-GPE interface. The MSD analysis of the Li^+^ (Fig. S6b) showed that the pristine LPSCl shows higher Li^+^ mobility compared with the pristine GPE. Notably, Li^+^ mobility in the hetero-phase electrolyte (i.e., the LPSCl-GPE interface) was substantially reduced compared with the single-phase electrolytes. This theoretical analysis was consistent with the ionic resistance result shown in Fig. 1c. We also theoretically calculated the *D*_Li+_ values of the different electrolytes (Fig. 1e). The pristine LPSCl showed the highest *D*_Li+_ (0.65 Å^2^ ns^−1^), followed by the LPSCl (0.27 Å^2^ ns^−1^) in the hetero-phase electrolyte, the pristine GPE (0.13 Å^2^ ns^−1^), and the GPE (0.10 Å^2^ ns^−1^) in the hetero-phase electrolyte. These results demonstrate that the Li^+^ conduction across the LPSCl-GPE interface was significantly retarded, compared with the pristine LPSCl and GPE.

### Effect of GPE Elasticity on Bi-percolating Ion Channels Formation of the CSE

In order to form bi-percolating ion channels of the LPSCl and GPE phases in the CSE, particular attention should be devoted to the GPE elasticity, as well as the composition ratio of the CSE described above. In this study, GPE elasticity was adjusted by varying the ETPTA polymer content. The Young’s modulus (E) values of the pristine GPEs tended to increase with the ETPTA polymer content (Fig. S7). For example, the ETPTA polymer content of 3.5 and 15 wt.% led to GPEs with low E (30 MPa, denoted as GPE-30) and high E values (220 MPa, denoted as GPE-220), respectively. When subjected to an environmental pressure of 74 MPa, the GPE-220 maintained its structural integrity, while the GPE-30 was severely disrupted (Fig. S8).

**Fig. 2:**
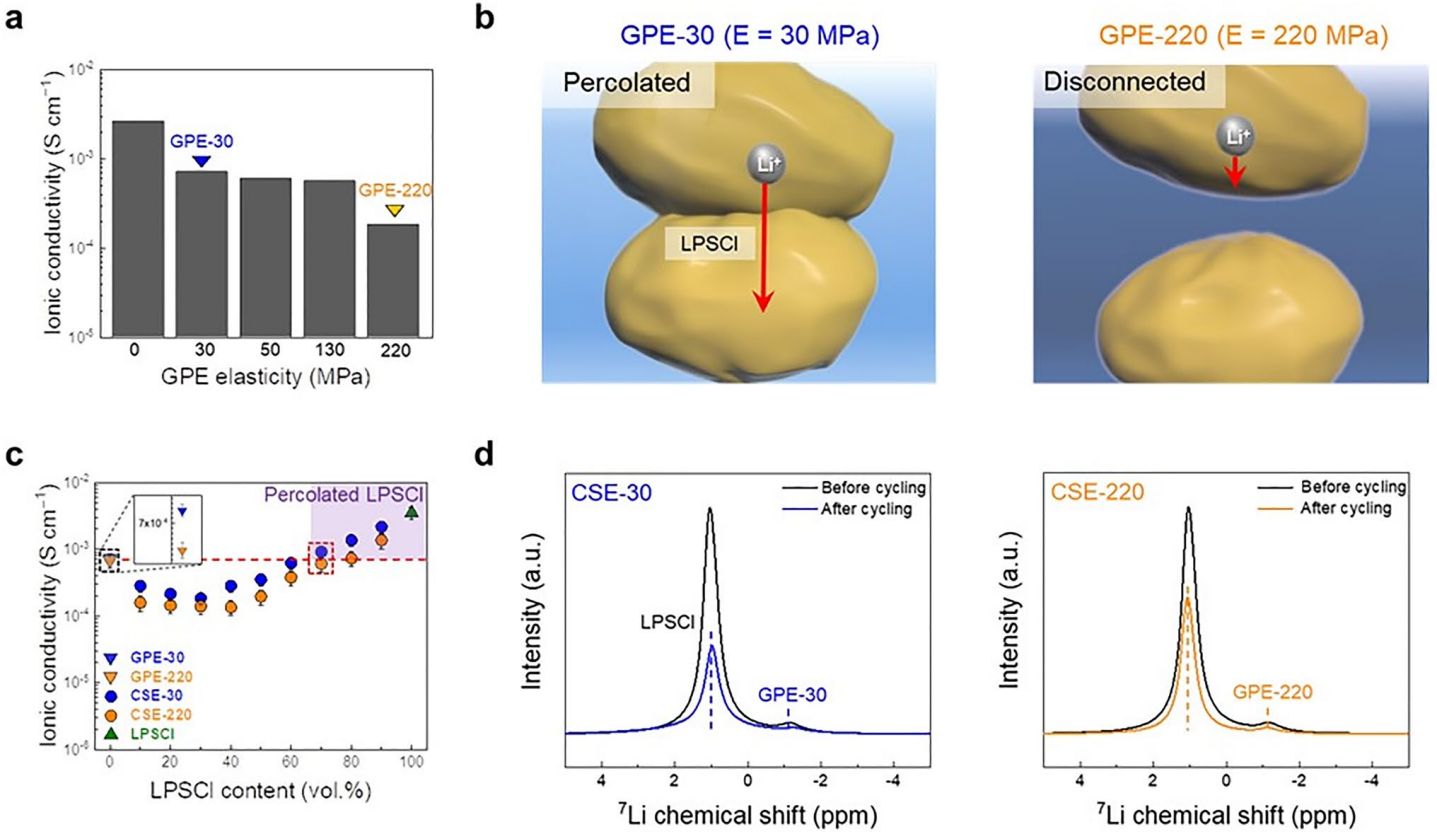
Effect of GPE elasticity on bi-percolating ion channel formation of the CSE. **a** Ionic conductivity of the CSE (LPSCl/GPE = 70/30 (v/v)) as a function of its GPE elasticity under a constant environmental pressure of 74 MPa and a temperature of 25 °C. **b** Schematic illustration depicting the dependence of the LPSCl percolation on the GPE elasticity (GPE-30 vs. GPE-220). **c** Ionic conductivity of the CSE with different GPE elasticities (GPE-30 vs. GPE-220) as a function of LPSCl content under a constant environmental pressure of 74 MPa and a temperature of 25 °C. The ionic conductivities of the samples were repeatedly measured three times to ensure the data reliability. **d** MAS ^7^Li NMR spectra of the Li||Li symmetric cells (^6^Li|CSE|.^6^Li) before and after the cycling test (900 min): CSE-30 vs. CSE-220

The effect of GPE elasticity on the ionic conductivity of the CSEs was investigated at a fixed composition ratio of LPSCl/GPE = 70/30 (v/v) under a constant environmental pressure of 74 MPa and a temperature of 25 °C. Figure 2a shows that higher GPE elasticity led to a reduction in the ionic conductivity of the CSE, indicating that the elastic GPE matrix may impede physical contact between the LPSCl particles, thus making it difficult to form the percolating channels of the LPSCl phase. This dependence of LPSCl percolation on GPE elasticity is conceptually illustrated in Fig. 2b.

To better understand this result, the ionic conductivities of the CSEs with different GPE elasticities (GPE-30 and GPE-220) were compared as a function of LPSCl content (Fig. 2c). Over the whole LPSCl/GPE composition ratios, the CSE (referred to as CSE-30) with the GPE-30 showed higher ionic conductivity than the CSE (CSE-220) with the GPE-220. As an extreme case, the LPSCl was mixed with the liquid-state LiG3 (i.e., without the ETPTA polymer), whereupon the ionic conductivity of the obtained LPSCl/LiG3 mixture began to increase even at lower LPSCl volume (30 vol.%, Fig. S9) compared the LPSCl/GPE systems (70 vol.%), indicating that the LPSCl phase was easily percolated owing to the fluidic characteristic of the LiG3 phase. By contrast, the ionic conductivities of the pristine GPEs (GPE-30 and GPE-220) were barely affected by the elasticity (0.71 mS cm^−1^ for GPE-30 and 0.67 mS cm^−1^ for GPE-220, as shown at 0 vol.% LPSCl content, Fig. 2c). This result exhibits that the higher ionic conductivity of the CSE-30 over a wide range of LPSCl content could be attributable to the well-developed percolating ion channels of the LPSCl phase.

The relationship between ion conduction pathways of the CSEs and their phase morphologies was elucidated by magic angle spinning (MAS) ^7^Li NMR analysis of symmetric cells (^6^Li|CSE|^6^Li, Figs. 2d and S10). The *ex-situ* MAS ^7^Li NMR spectra of the CSE showed a reduction in the peak intensities of the LPSCl (at 1.05 ppm) and GPE (at − 1.17 ppm) phases after the cycling test (900 min), revealing that ^7^Li (originating from the LPSCl and GPE in the CSE) was partially replaced by ^6^Li (from the ^6^Li metal electrodes). Furthermore, the decrease in the peak area ratio of the LPSCl phase was noticeable at the CSE-30 (9.9 → 5.3), compared with the CSE-220 (6.9 → 6.8) (Table S2), verifying the formation of highly percolated LPSCl channels. Simultaneously, the CSE-30 showed a higher Li^+^ transference number (*t*_Li+_  = 0.69) than the CSE-220 (*t*_Li+_  = 0.37, Fig. S11), underlying the advantageous contribution of the percolated LPSCl phase that is an intrinsic single Li^+^ conductor (*t*_Li+_  = 1) [12]. These results demonstrate how GPE elasticity is important in establishing the percolating ion channels of the LPSCl phase in the CSE.

**Fig. 3:**
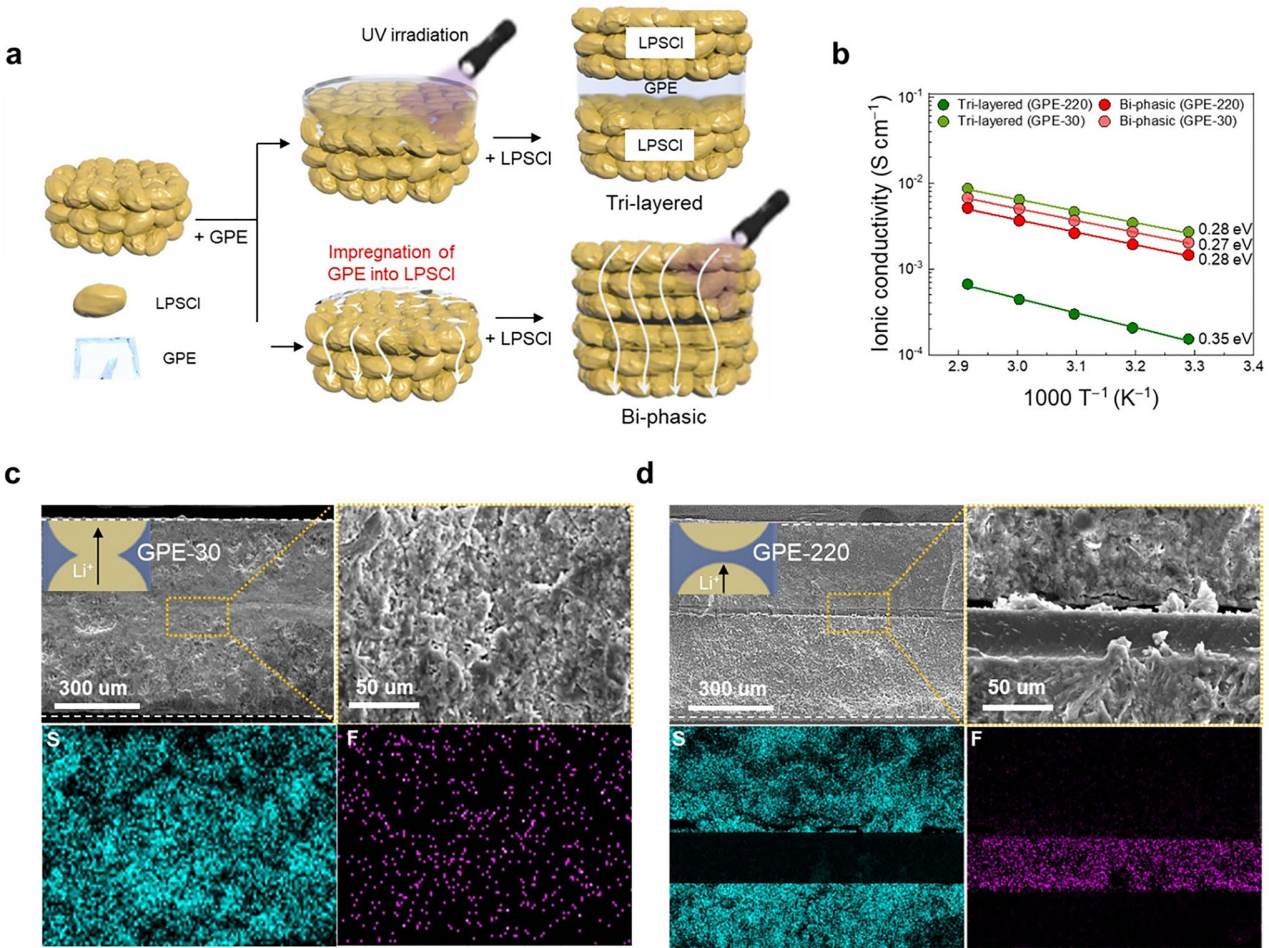
Comparison between tri-layered and bi-phasic CSEs. **a** Schematic illustration depicting the stepwise fabrication procedure of the two model CSEs (tri-layered vs. bi-phasic). **b** Temperature-dependent ionic conductivity of the two model CSEs (tri-layered vs. bi-phasic) under a constant environmental pressure of 74 MPa. Cross-sectional SEM and EDS elemental mapping images (F (originating from the GPE) and S elements (from the LPSCl)) of the tri-layered CSEs: **c** CSE-30 and **d** CSE-220

To highlight the effect of GPE elasticity in forming the bi-percolating channels of the CSE, we prepared tri-layered and bi-phasic CSEs with a fixed LPSCl content of 95 vol.% as model systems. Details on the composition ratios of the model systems were described in Table S3. To prepare a tri-layered (LPSCl-GPE-LPSCl) CSE, a self-standing LPSCl layer was first fabricated at a constant environment pressure of 74 MPa. At the same time, a self-standing GPE layer was independently fabricated through UV irradiation-assisted curing of a GPE precursor (i.e., LiG3/ETPTA monomer). Subsequently, the UV-cured self-standing GPE layer (thickness ~ 30 μm) was placed between the two self-standing LPSCl layers (thickness ~ 300 μm) and followed by pressing at 74 MPa, eventually producing the tri-layered CSE. For the preparation of the bi-phasic CSE, self-standing LPSCl layers were first fabricated using the same procedure described above. Onto these LPSCl layers (porosity = 9.1%, Table S4), the liquid-state GPE precursor was dropped and stored to allow its infiltration into the pores (i.e., interstitial voids between the LPSCl particles) of the LPSCl layers in the through-thickness direction. Sequentially, the GPE precursor-embedded two LPSCl layers were sandwiched to face each other and subjected to the pressing at 74 MPa followed by UV irradiation to crosslink the ETPTA monomer of the GPE, eventually producing the bi-phasic CSE. The porosity (9.1%) of the LPSCl layers was reduced to ~ 5.2% after the introduction of the GPE (Table S4), exhibiting the successful infiltration of the GPE into the pores of the LPSCl layers. The stepwise fabrication procedure of the two model CSEs is schematically depicted in Figs. 3a and S12.

Both the temperature-dependent ionic conductivity and activation energy (*E*_a_) of the model CSEs were investigated as a function of GPE elasticity under a fixed environmental pressure of 74 MPa (Fig. 3b). For the GPE-30, no significant difference in the ionic conductivity and activation energy was observed between the tri-layered (σ = 0.24 mS cm^−1^ and *E*_a_ = 0.28 eV) and bi-phasic (σ = 0.25 mS cm^−1^, *E*_a_ = 0.27 eV) CSEs, indicating that the percolating ion channel of the LPSCl phase was well-formed in the two model CSEs. This result exhibits that the GPE-30 layer in tri-layered CSE, owing to its low elasticity, could infiltrate into the pores (i.e., interstitial voids between the LPSCl particles) of the LPSCl layers upon being subjected to the pressing (Table S5). By contrast, the model CSEs with the GPE-220 showed different ion conduction behavior. The tri-layered CSE demonstrated markedly lower ionic conductivity (σ = 0.016 mS cm^−1^) and higher activation energy (*E*_a_ = 0.35 eV) compared with the bi-phasic CSE (σ = 0.14 mS cm^−1^ and *E*_a_ = 0.28 eV). This result indicates that due to its high elasticity the GPE-220 middle layer in the tri-layered CSE may inhibit physical contact between the top and bottom LPSCl layers, thus preventing the formation of percolating ion channels of the LPSCl.

This result was further verified by analyzing cross-sectional SEM and EDS elemental mapping images of the tri-layered CSEs. For the CSE with the GPE-30, the LPSCl particles were highly interconnected (Fig. 3c). Furthermore, fluorine (F) (originating from the GPE) and sulfur (S) (originating from the LPSCl) were uniformly distributed in the through-thickness direction of the CSE. By comparison, the GPE-220 layer in the CSE maintained its structural integrity, indicating that the top and bottom LPSCl layers were physically isolated (Fig. 3d). This model study demonstrates that GPE elasticity plays a viable role in forming the bi-percolating ion channels of the LPSCl and GPE phases in the CSE.

### Elucidating Ion Transport across the LPSCl-GPE Interface

The ion conduction mechanism of the LPSCl is entirely different from that of the GPE: Li^+^ diffusion through interstitial sites [3] (for the LPSCl) and migration of Li^+^-glyme complexes [38] (for the GPE), respectively. Thus, a mechanistic understanding of ion transport across the LPSCl-GPE interface should be considered as a prerequisite before developing CSEs.

**Fig. 4:**
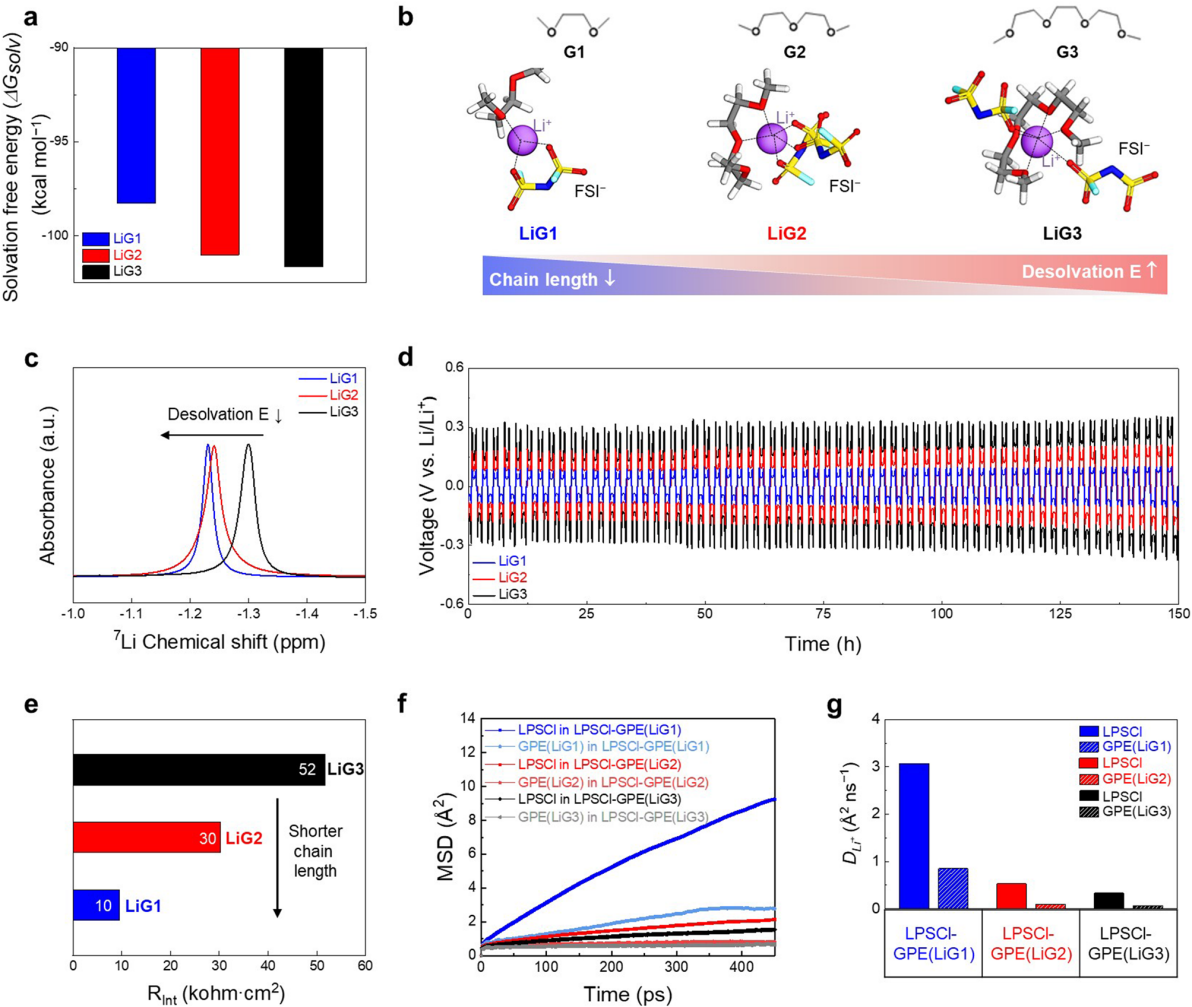
Elucidating ion transport across the LPSCl-GPE interface. **a** Solvation free energy (∆G_solv_) of the different Li^+^-glyme complexes (LiG1, LiG2, and LiG3). **b** Comparison in the solvation structure of the different Li^+^-glyme complexes with a focus on Li–O coordination number. **c** ^7^Li NMR spectra of the different Li^+^-glyme complexes. **d** Voltage profiles of Li||Li symmetric cells containing the different Li^+^-glyme complexes at a current density of 1 mA cm^−2^ and plating/stripping capacity of 1 mAh cm^−2^. **e** Interfacial resistance (*R*_Int_, corresponding to ion transport across the LPSCl-GPE interface) of the different Li^+^-glyme complexes. **f** Mean square displacement (MSD) of the Li^+^ in the hetero-phase electrolytes (i.e*.*, across the LPSC-GPE interface) in the CSE, in which the GPE contained the different Li^+^-glyme complexes (LiG1, LiG2, and LiG3) **g** Li^+^ diffusion coefficients (*D*_Li+_) across the LPSCl-GPE interface as a function of chain length of glyme. Note that the Figure legends represent the constituents in the hetero-phase electrolytes

Firstly, we theoretically elucidated the solvation/desolvation behavior of equimolar Li^+^-glyme complexes in the GPEs. The solvation free energies of the Li^+^-glyme complexes were calculated as a function of the chain length of glyme molecules (i.e., GX (X = 1 (monoglyme), 2 (diglyme), and 3 (triglyme)) using MD simulation (Fig. S13, see the Experimental Section). The LiG1 showed a less negative value of solvation free energy (*∆G*_*solv*_ =  − 98.3 kcal mol^−1^) compared with other LiGX (LiG2 =  − 101.03 and LiG3 =  − 101.66, Fig. 4a), indicating that the desolvation of solvated Li^+^ in the LiG1 is thermodynamically favorable. This difference in the solvation free energy in the Li^+^-glyme complexes can be attributable to changes in the Li^+^ coordination structure. We analyzed the Li^+^ coordination number of the Li^+^-glyme complexes using radial distribution function (RDF) analysis (Fig. S14). Shorter glyme chain lengths (corresponding to the lower number of oxygen) resulted in a decrease in the Li–O coordination number (Fig. 4b), which accounts for the lower solvation energy in the LiG1.

This theoretical elucidation of the solvation/desolvation behavior was experimentally verified by analyzing ^7^Li NMR spectra as a function of the glyme chain length. Figure 4c shows that the shorter chain length of the glymes led to a downfield shift in the singlet ^7^Li peak, indicating the enhanced dissociation of the LiFSI salt [40]. This result is consistent with the theoretical result of the solvation/desolvation energy shown in Fig. 4a. In order to provide additional evidence, the effect of chain length of the glymes on Li plating/stripping cyclability was examined using Li||Li symmetric cells at a current density of 1 mA cm^−2^ and a plating/stripping capacity of 1 mAh cm^−2^ (Fig. 4d). Both LiG2 and LiG3 showed significant voltage fluctuations along with large overpotentials, which were more severe in the LiG3 as a result of having a longer chain length. By comparison, the LiG1 showed the most stable cycling performance. This result was verified by conducting the EIS analysis after the cycling test (Fig. S15). Cell resistance increased as the chain length of the glymes became longer. Notably, both interfacial resistance (*R*_Int_) and charge transfer resistance (*R*_CT_) tended to increase as the chain length of the glymes increased from G1 to G3, while the bulk resistance (*R*_Bulk_) remained almost unchanged. This result demonstrates that the chain length of the glymes plays a significant role in regulating the solvation/desolvation phenomena of the Li^+^-glyme complexes.

Based on this understanding of the equimolar Li^+^-glyme complexes in the GPEs, we investigated their effect on ion transport across the LPSCl-GPE interfaces with a focus on the design of the GPE chemistry, in which the composition ratio of LiGX (X = 1, 2, and 3)/ETPTA in the GPE was set to 96.5/3.5 (w/w) under the condition that the bi-percolating ion channels of the LPSCl and GPE phases were already formed in the CSE. The LiG1 and LiG2 were chemically stable upon contact with the LPSCl (Fig. S16), which was a similar to the result to the LiG3 (Fig. S1). Prior to conducting an in-depth investigation of the ion transport across the LPSCl-GPE interface, we measured the bulk resistance of the CSEs as a function of the glyme chain length (Fig. S17). The CSE with the LiG1-containing GPE (denoted as CSE (LiG1)) showed the lowest bulk resistance compared to the other CSEs (CSE (LiG2) and CSE (LiG3)). From the previous result shown in Fig. S15, we found that the bulk resistance of the glymes themselves (LiG1, LiG2, and LiG3) was negligibly dependent on their chain length. Thus, this lowest bulk resistance (accounting for the highest ionic conductivity) of the CSE (LiG1) indicates that Li^+^ diffusion across the LPSCl-GPE (including LiG1) interface could be facilitated. To further elucidate this result, ion transport across the LPSCl-GPE interface was examined as a function of the chain length of glymes (Fig. S18) using the same EIS analysis technique shown in Fig. 1c. The GPE with the LiG1 showed the lowest interfacial resistance (*R*_Int_) of 10 kΩ cm^2^ compared to other GPEs with the LiG2 (30 kΩ cm^2^) and LiG3 (52 kΩ cm^2^) (Fig. 4e).

These results were consistent with the easier ionic dissociation (indicated by the lower desolvation energy described in Fig. 4b) of the LiG1 and were further verified by theoretically examining Li^+^ conduction behavior using MSD analysis across the LPSCl-GPE interface model systems (Fig. S19) as a function of the chain length of glymes. The MSD across the LPSCl-GPE interface was increased in the order of LiG3, LiG2, and LiG1 in the GPEs (Fig. 4f). To better understand this intriguing behavior, Li^+^ diffusion coefficients (*D*_Li+_) across the LPSCl-GPE interface were calculated as a function of glyme chain length (Fig. 4g): *D*_Li+_ in the LPSCl phase are LiG1 = 3.07, LiG2 = 0.53, and LiG3 = 0.33 Å^2^ ns^−1^, while *D*_Li+_ in the GPE phase are LiG1 = 0.85, LiG2 = 0.10, and LiG3 = 0.07 Å^2^ ns^−1^. This result shows that the GPE with the LiG1 facilitates Li^+^ diffusion across the LPSCl-GPE interface compared to other GPEs with LiG2 and LiG3, underscoring the significant role of the desolvation free energy (that was strongly affected by glyme chain length) in the GPEs.

### Enabling the Development of High-energy–density SSB Full Cells using the CSE

**Fig. 5:**
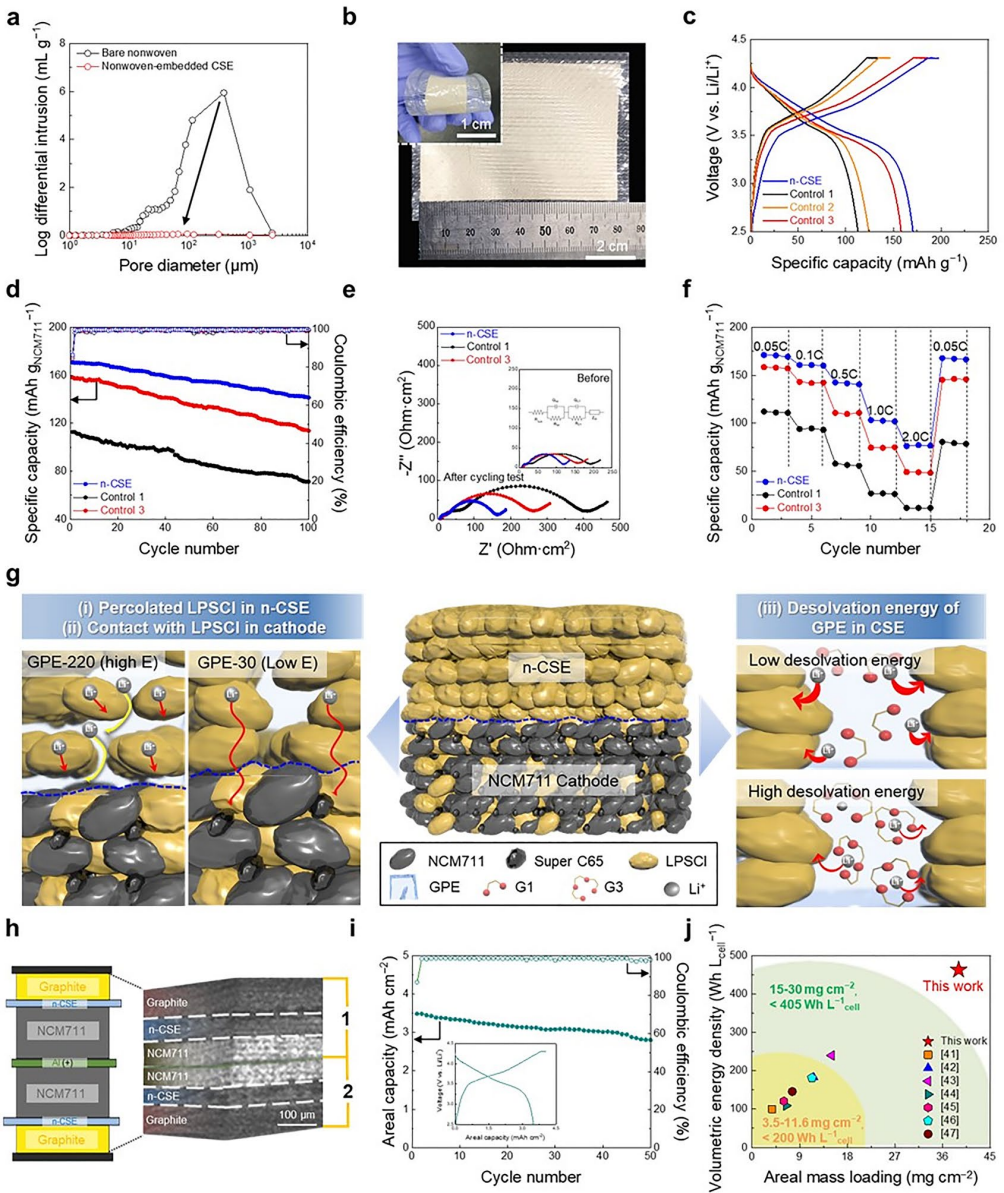
Enabling practical high-energy–density SSB full cells by the CSE. **a** Pore size distribution of the pristine nonwoven substrate and n-CSE (LPSCl/GPE-30 (containing the LiG1)). **b** Photograph of the n-CSE (length × width = 80 × 60 (mm × mm)). Inset shows the mechanical flexibility of the n-CSE upon bending deformation. **c** Voltage profiles (at 1st cycle) of the pellet-type SSB full cell with the n-CSE (vs. control composite membranes 1, 2, and 3) at an operating temperature of 25 °C. **d** Cycling performances of the pellet-type SSB full cell with the n-CSE (vs. control composite membranes 1 and 3) at an operating temperature of 25 °C. **e** EIS profiles of the SSB full cells with the n-CSE (vs. control composite membranes 1 and 3) before (inset) and after the cycling test. **f** Discharge rate capability of the SSB full cells with the n-CSE (vs. control composite membranes 1 and 3). **g** Conceptual illustration of the SSB full cells with the n-CSE and control composite membranes, with a focus on the percolated LPSCl in the n-CSE, its close contact with the LPSCl in the cathode, and the desolvation energy of the GPE phase in the n-CSE. **h** Schematic representation and X-ray CT image of the pellet-type SSB full cell with the bi-cell configuration (one double-side-coated NCM711 cathode (areal-mass-loading = 39 mg cm^–2^) assembled with two graphite anodes and two n-CSE membranes). **i** Cycling performance of the SSB full cell with the bi-cell configuration at an operating temperature of 25 °C. The inset shows the 1^st^ charge/discharge profile. **j** Comparison of the volumetric energy density (plotted as a function of areal-mass-loading of cathode) between the n-CSE-based SSB (this study) and previously reported CSE-based SSBs

To enable the development of practical high-energy–density SSB full cells, it is preferable to use thin, flexible, and scalable solid-state electrolytes that can ensure facile ion conduction along with intimate interfacial contact with electrodes. As a practical approach to achieve this goal, the CSE (LPSCl/LiG1-containing GPE) was integrated with an aramid nonwoven porous substrate acting as a mechanically compliant scaffold (thickness ~ 18 μm, porosity ~ 86%, Fig. S20). The resulting nonwoven-embedded CSE (denoted as n-CSE) showed no appreciable levels of pores (Fig. 5a), revealing that the nonwoven pores were almost completely filled with the CSE. The obtained n-CSE showed high ionic conductivity (σ = 0.41 mS cm^−1^ at 25 °C, Fig. S21), which was inappreciably different from that of the pristine CSE (σ = 0.76 mS cm^−1^). The CSE components in the n-CSE remained almost undetached after the peel-off test (Fig. S22), indicating the structural robustness of the n-CSE.

Introducing the nonwoven scaffold allowed the n-CSE to show improvements in the thickness (~ 40 µm) and area (length × width = 80 × 60 (mm × mm)), underscoring its practical viability and manufacturing scalability (Fig. 5b). Moreover, the n-CSE maintained its electrochemical activity after 100 folding cycles (Fig. S23). We note that these physicochemical properties of the n-CSE would be difficult to achieve using conventional inorganic solid electrolytes. The control n-CSE membranes were prepared with three different control composite membranes: control 1 (LiG3, 220 MPa) consisting of LPSCl and GPE1 (LiG3 and 15 wt.% ETPTA), control 2 (LiG1, 220 MPa) consisting of LPSCl and GPE2 (LiG1 and 15 wt.% ETPTA), and control 3 (LiG3, 30 MPa) consisting of LPSCl and GPE3 (LiG3 and 3.5 wt.% ETPTA). The ionic conductivities of the control composite membranes were 0.14 (control 1), 0.17 (control 2), and 0.23 (control 3) mS cm^−1^ (Fig. S24).

The n-CSE was assembled with an NCM711 cathode (NCM711/LPSCl/super C65/polybutadiene rubber binder = 74.5/21.5/2/2 (w/w/w/w), areal capacity = 1.75 mAh cm^−2^) and a graphite anode (graphite/LPSCl/polybutadiene rubber binder = 75/22/3 (w/w/w), areal capacity = 1.93 mAh cm^−2^) in order to fabricate an SSB full cell (N/P ratio = 1.1), in which the fabrication details are described in the Experimental Section. Figure 5c shows the charge/discharge voltage profiles of the SSB full cells at the 1^st^ cycle using a voltage range of 2.5–4.3 V. The n-CSE presented stable voltage profiles along with a normal discharge capacity. By comparison, the control composite membranes showed greater cell polarization and lower discharge capacities, which were much more pronounced in control composite membrane 1 containing the GPE-220.

The n-CSE showed higher cycling retention (= 82.9% after 100 cycles) compared with the control composite membranes (Fig. 5d) at 25 °C. This superior cyclability of the n-CSE was verified by analyzing the EIS spectra after the cycling test. The SSB full cells with the control composite membranes showed a marked increase in cell impedance (Fig. 5e and Table S6). By contrast, the SSB full cell with the n-CSE suppressed the growth of cell impedance after the cycling test. In addition to better cyclability, the SSB full cell with the n-CSE showed higher discharge capacities over a wide range of discharge current densities (0.05 C–2.0 C) compared with the control composite membranes (Fig. 5f).

In this study, the electrodes of the SSB full cells contained only LPSCl (without the GPE), while the n-CSE membrane consisted of both LPSCl and GPE. Considering the sluggish ion conduction across the LPSCl-GPE interfaces (as shown in Figs. 1 and 4), the LPSCl phase in the n-CSE membrane should be in close contact with the LPSCl in the electrodes to ensure facile ion transport at the CSE membrane-electrode interfaces. Compared to the n-CSE membrane, the control composite membranes 1 and 2 presented the poorly interconnected channels of the LPSCl phase due to the GPE-220 with high elasticity. Consequently, it was more difficult for the LPSCl phase in control composite membranes 1 and 2 to establish intimate contact with the LPSCl in the electrode, resulting in the low cell performance. This comparison with the control composite membranes 1 and 2 showed that the LPSCl percolation is critical to achieve the high ionic conductivity of the n-CSE and the good contact with the LPSCl in the electrode, which eventually affects the cell performance. Meanwhile, despite the presence of the percolated LPSCl enabled by the GPE-30 with low elasticity, the control composite membrane 3 showed poor cell performance due to the high desolvation energy of LiG3 in the GPE, exhibiting the significant role of the desolvation energy in the cell performance. From this result, we found that the control composite membrane 1 showed the lowest cell performance due to the combined effect of the poorly interconnected channels of the LPSCl phase and high desolvation energy of LiG3 in the GPE. The comparison with the control composite membranes is schematically depicted in Fig. 5g, which demonstrates that (i) the percolated LPSCl in the n-CSE, (ii) its close contact with the LPSCl in the cathode, and (iii) the desolvation energy of the GPE phase in the n-CSE play important roles in achieving a reliable electrochemical performance for the SSB full cells.

To develop a high-energy–density SSB full cell, a bi-cell configuration was used (Fig. 5h). A double-side-coated NCM711 cathode (areal-mass-loading = 39 mg cm^–2^) was assembled with two graphite anodes and two n-CSE membranes. The well-defined bi-cell structure, with intimate interfacial contact between the component layers, was verified by imaging the SSB full cell using X-ray computed tomography (CT). The resulting bi-cell showed a normal charge/discharge profile after the 1^st^ cycle and a high areal capacity of 3.5 mAh cm^−2^ (inset of Fig. 5i). Notably, the bi-cell achieved high volumetric energy densities (= 480 Wh L_cell_^−1^) at 25 °C, where the energy densities were estimated based on electrode volume [38, 41–47] (including current collectors) and n-CSEs (see Table S7 for calculation details). In addition to the high-energy density, a stable cycling retention (~ 80.4% after 50 cycles) was observed in the bi-cell (Fig. 5i). Our future work will aim to further advance CSE chemistry and optimize cell design to achieve high-energy–density SSB full cells with enhanced longevity.

A salient achievement of this SSB bi-cell is that it can provide high volumetric energy density (= 480 Wh L_cell_^−1^) at 25 °C while fulfilling the challenging requirements of the high areal-mass-loading of the cathode (= 39 mg cm^−2^) and N/P ratio (= 1.1), which massively outperformed those of the previously reported CSE-based SSB full cells (Fig. 5j). Details (including the electrode information and cell operating conditions) on the comparison between this study and previous reports are presented in Table S8. This comparative study demonstrates that the CSE-based bi-cell configuration can be suggested as a promising approach for facilitating the development of a practical high-energy–density SSB full cell.

## Conclusions

In summary, we have elucidated the ion transport phenomena in the CSEs with a focus on bi-percolating ion channel formation and ion conduction across LPSCl-GPE interfaces. The percolation threshold of the LPSCl phase in the CSE was strongly dependent on the elasticity of the GPE phase. Manipulating the solvation/desolvation behavior of Li^+^-glyme complexes in the GPE phase facilitated ion conduction across the LPSCl-GPE interfaces. The optimal CSE that fulfilled the above-described electrolyte requirements was integrated with the nonwoven porous substrate to produce a thin and scalable n-CSE. The obtained n-CSE enabled the SSB full cell (NCM711 cathode/graphite anode) with bi-cell configuration to exhibit high volumetric energy density (480 Wh L_cell_^−1^) and stable cycling retention at 25 °C under constrained cell conditions (areal-mass-loading of NCM711 cathode = 39 mg cm^–2^ (corresponding to the areal capacity of 3.5 mAh cm^−2^) and N/P ratio = 1.1), which lie far beyond those of previously reported CSE-based SSB full cells. We envision that this mechanistic understanding of the ion transport phenomena in the CSEs will provide a versatile knowledge platform for advanced CSE design encompassing various sulfide/polymer electrolyte mixtures and can be utilized in other emerging solid-state batteries based on post-Li chemistry.

### Supplementary Information

Below is the link to the electronic supplementary material.

Supplementary file1 (PDF 1562 KB)
