# Supplementary material for: Elucidating Ion Transport Phenomena in Sulfide/Polymer Composite Electrolytes for Practical Solid-State Batteries
Source: Nanomicro Lett. 2023 Jul 13;15:179. doi: 10.1007/s40820-023-01139-w (PMC10344856; doi:10.1007/s40820-023-01139-w)
Supplement: Supplementary file 1 — Supplementary file1 (PDF 1562 KB) [file 40820_2023_1139_MOESM1_ESM.pdf]

Nano-Mciro Letters

Supporting Information for

Elucidating Ion Transport Phenomena in Sulfide/Polymer Composite Electrolytes for Practical Solid-State Batteries

Kyeong-Seok Oh[1, †], Ji Eun Lee[3†], Yong-Hyeok Lee[1, †], Yi-Su Jeong[3], Imanuel Kristanto[2], Hong-Seok Min[4], Sang-Mo Kim[4], Young Jun Hong[4], Sang Kyu Kwak[2, *], and Sang-Young Lee[1, *]

[1] Department of Chemical and Biomolecular Engineering, Yonsei University, 50 Yonsei-ro, Seodaemun-gu, Seoul, 03722, Republic of Korea

[2] Department of Chemical and Biological Engineering, Korea University, 145 Anam-ro, Seongbuk-gu, Seoul 02841, Republic of Korea

[3] School of Energy and Chemical Engineering, Ulsan National Institute of Science and Technology (UNIST), 50 UNIST-gil, Eonyang-eup, Ulju-gun, Ulsan, 44919, Republic of Korea

[4] Hyundai Motor Company, 150, Hyundaiyeonguso-ro, Namyang-eup, Hwaseong-si, Gyeonggi-do, 18280, Republic of Korea

[†] Kyeong-Seok Oh, Ji Eun Lee, and Yong-Hyeok Lee equally contributed to this work.

*Corresponding authors. E-mail: skkwak@korea.ac.kr (S.K.K.), syleek@yonsei.ac.kr (S.-Y.L)

Supplementary Figures and Tables

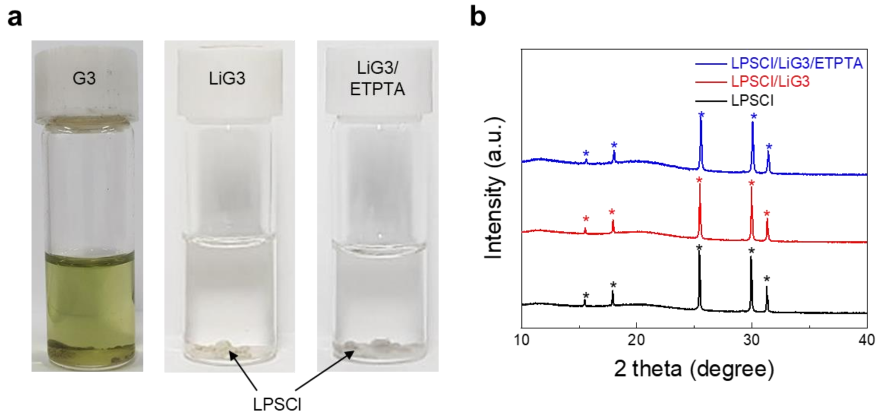

Fig. S1 Chemical stability of the CSE components after being kept for seven days. **a** Photographs and **b** XRD patterns in which the LPSCl was mixed with G3, LiG3 (LiFSI/G3 = 1/1 (mol/mol)), and GPE precursor (LiG3/ETPTA monomer = 85/15 (w/w))

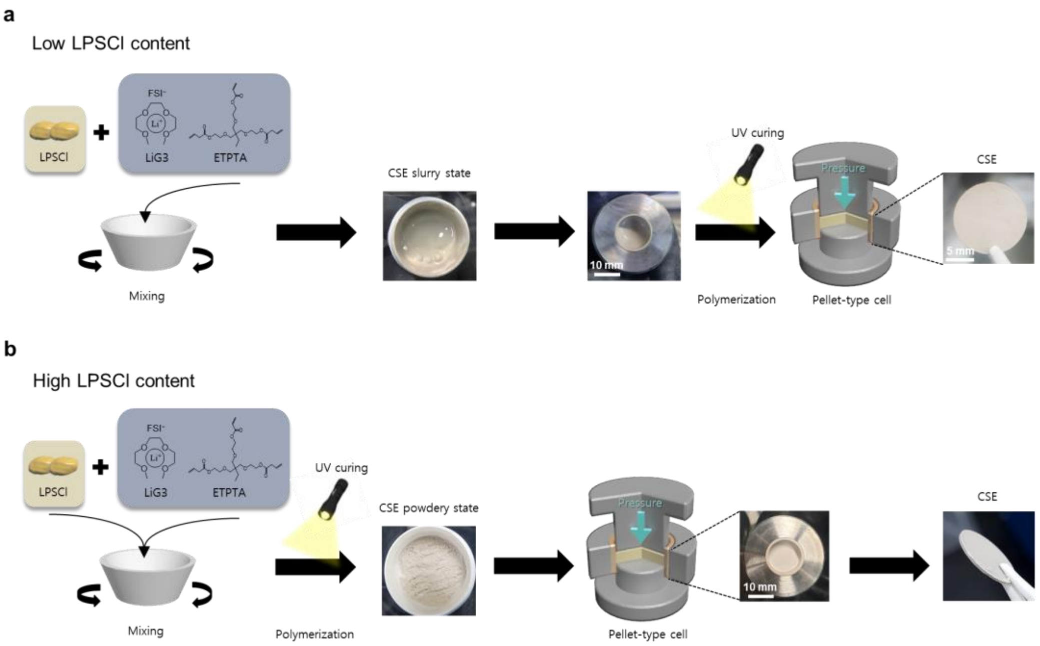

Fig. S2 Schematic representation depicting the stepwise fabrication procedure of the CSE, in which its chemical structure and photograph depicting CSEs (after the pressing at 74 MPa) was also provided. Depending on the LPSCl contents in the CSEs, the detailed fabrication processes of CSE pellets were adjusted as follows: **a** CSE slurry state (low LPSCl content (e.g., 10 vol.%)) and **b** CSE powdery state (high LPSCl content (e.g., 90 vol.%)) prior to the pressing

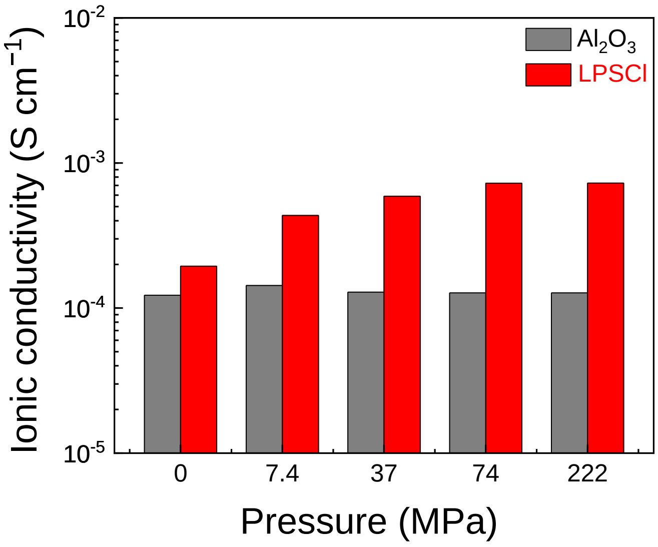

Fig. S3 Ionic conductivity of the CSE as a function of the applied pressure at a fixed composition ratio of LPSCl (and Al_2O_3)/GPE = 70/30 (v/v)

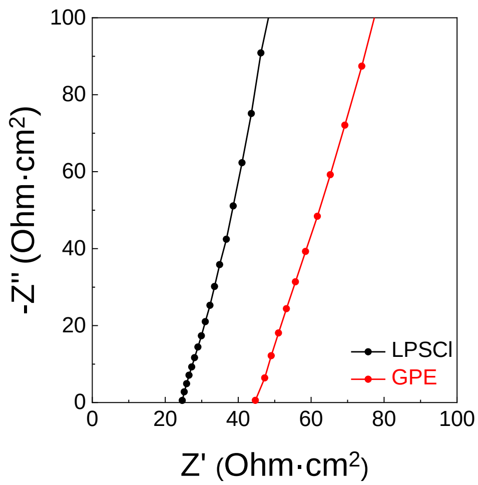

Fig. S4 Nyquist plots of the individual LPSCl and GPE layer

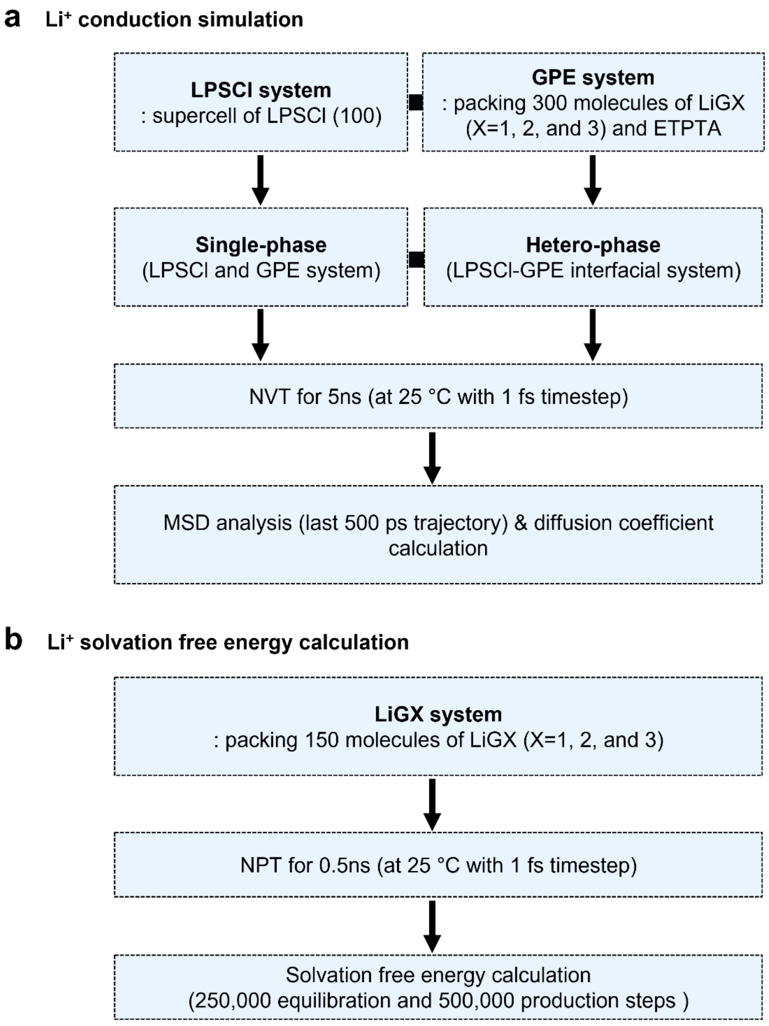

Fig. S5 Flow chart of MD simulation for **a** Li^+ conduction in the single- and hetero-phase system and **b** Li^+ solvation free energy calculation in the LiGX (X=1, 2, and 3) system

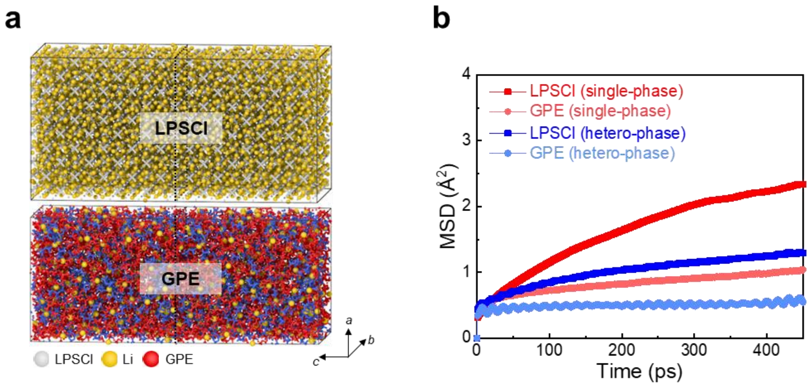

Fig. S6 a Model systems used to simulate Li$^+$ conduction through single-phase electrolytes (LPSCl and GPE, respectively) in the CSE. **b** Mean square displacement (MSD) of the Li$^+$ inside the single-phase (LPSCl and GPE) and hetero-phase electrolytes (i.e., across the LPSC-GPE interface) in the CSE

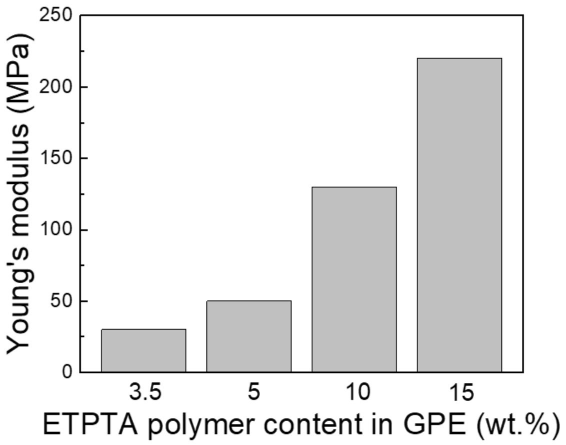

Fig. S7 Young's modulus of the pristine GPEs as a function of ETPTA polymer content

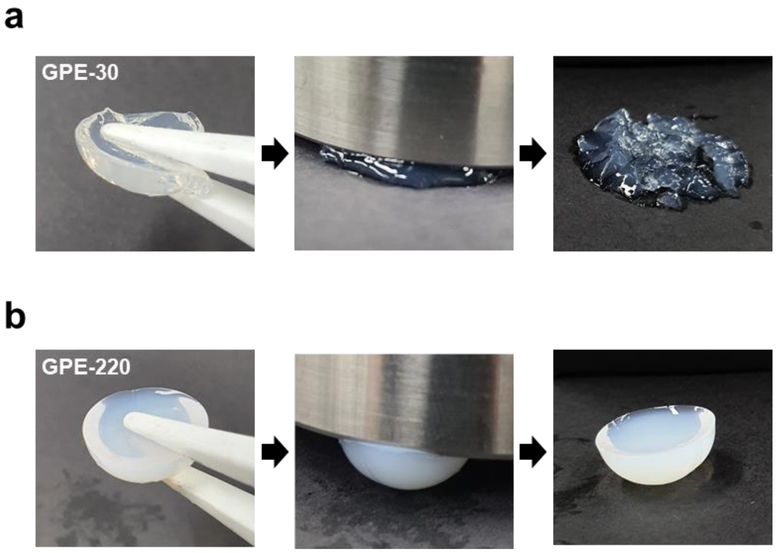

Fig. S8 Photographs of **a** GPE-30 and **b** GPE-220 upon being subjected to an environmental pressure of 74 MPa

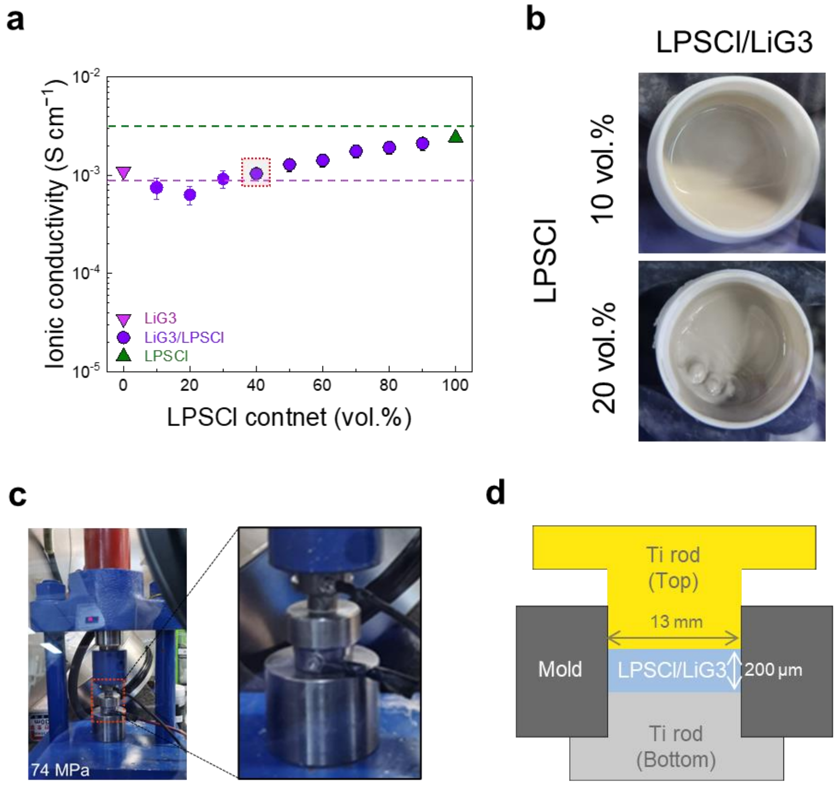

Fig. S9 **a** Ionic conductivity of the LPSCl/LiG3 as a function of LPSCl content. The ionic conductivities of the samples were repeatedly measured three times to ensure the data reliability. **b** Photographs of the LPSCl/LiG3 (LPSCl content = 10 and 20 vol.%) slurries. **c** Photographs of the LPSCl/LiG3 (LPSCl content = 10 vol.%) upon being subjected to an environmental pressure of 74 MPa. **d** Schematic illustration of the ionic conductivity measurement for the samples with low LPSCl contents

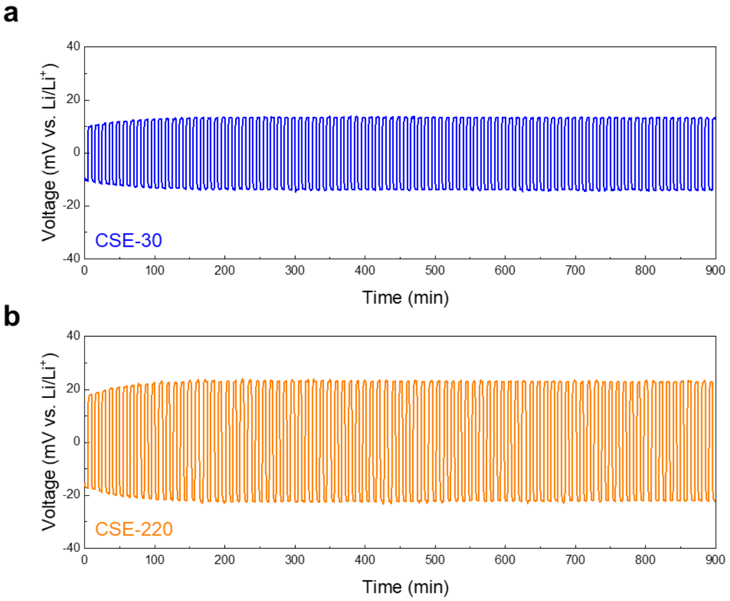

Fig. S10 Voltage profiles of the ^{6}Li||^{6}Li symmetric cell at a current density of 50 μA cm^{-2} and a plating/stripping capacity of 8.3 μAh cm^{-2}: **a** CSE-30 and **b** CSE-220

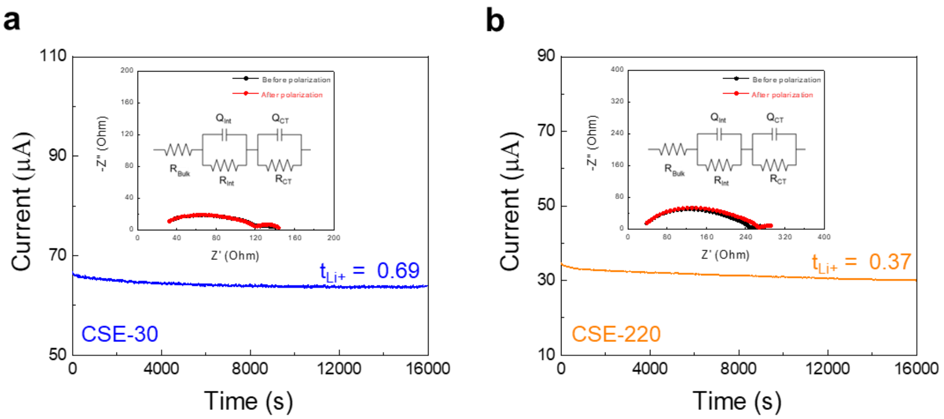

Fig. S11 Time-dependent current profiles and EIS profiles (inset) of the Li||Li symmetric cells at 10 mV polarization: **a** CSE-30 and **b** CSE-220

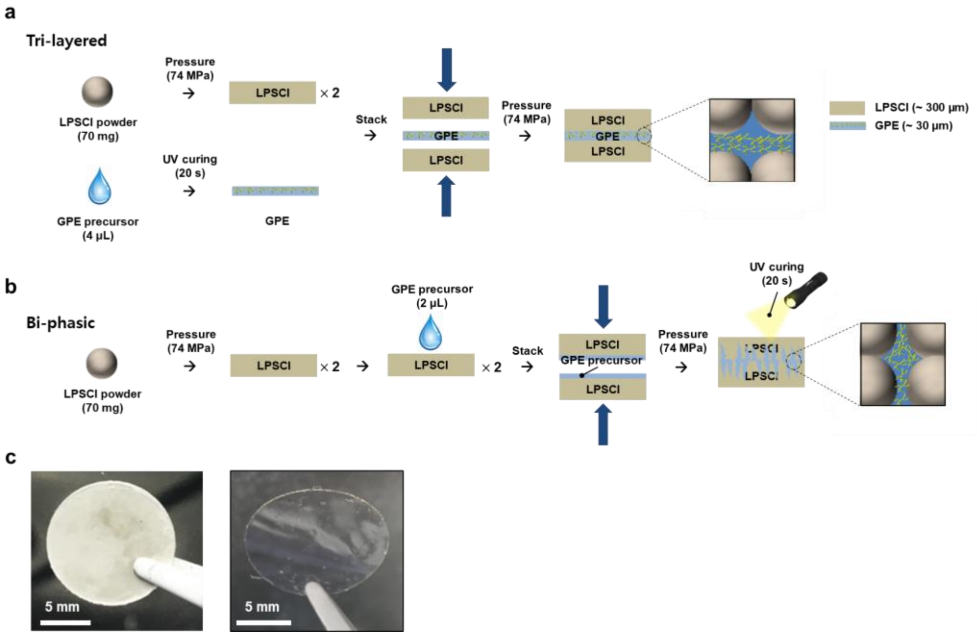

Fig. S12 Schematic illustration depicting the stepwise fabrication procedure of the two model CSEs: **a** tri-layered and **b** bi-phasic systems. **c** Photographs of the self-standing LPSCl and GPE layers used for the model CSEs

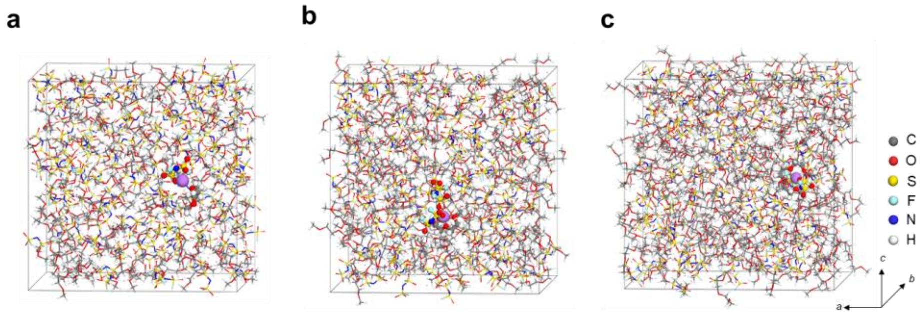

Fig. S13 Model systems used to simulate solvation of Li^+-glyme complexes as a function of glyme chain length: **a** LiG1, **b** LiG2, and **c** LiG3

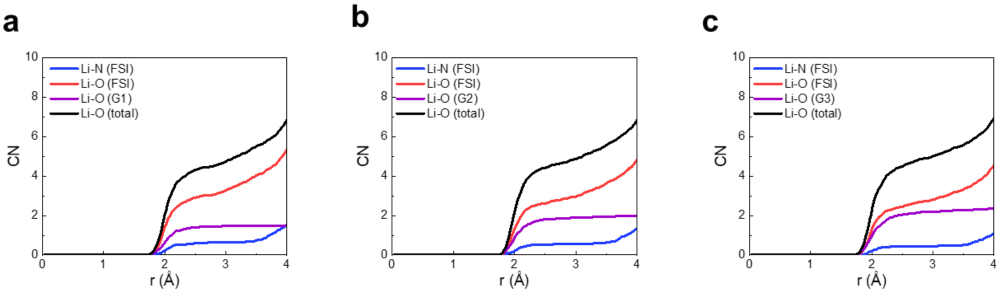

Fig. S14 Li^+ coordination number of the Li^+-glyme complexes obtained by radial distribution function (RDF) analysis: **a** LiG1, **b** LiG2, and **c** LiG3

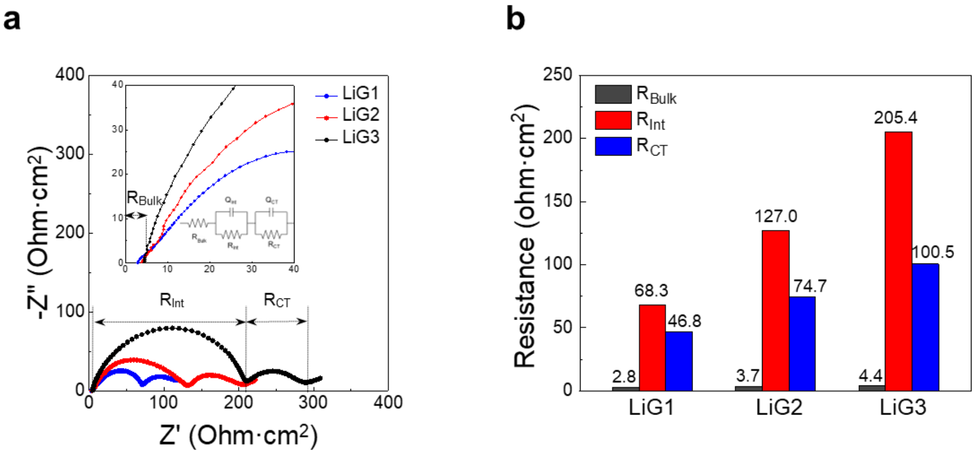

Fig. S15 a EIS spectra after the cycling test and **b** comparison in the R_{Bulk}, R_{Int}, and R_{CT} of the Li||Li symmetric cells with different Li^+-glyme complexes (LiG1, LiG2, and LiG3)

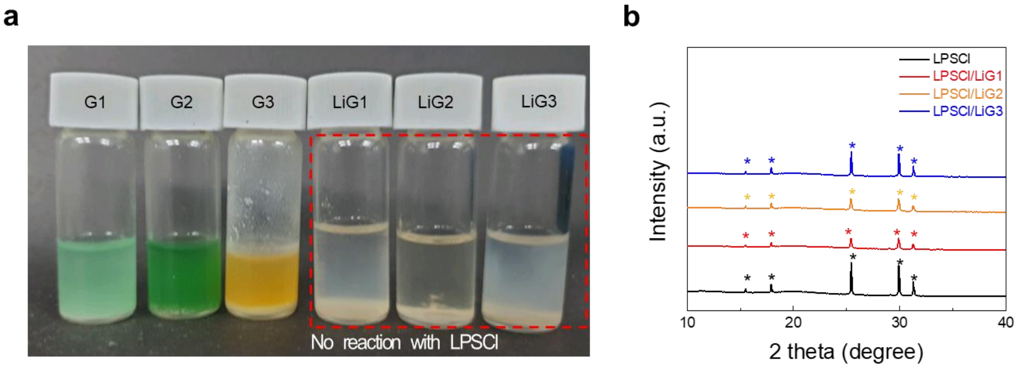

Fig. S16 Chemical stability of the LiGX (X = 1, 2, and 3) with the LPSCl after being kept for seven days. **a** Photographs and **b** XRD patterns

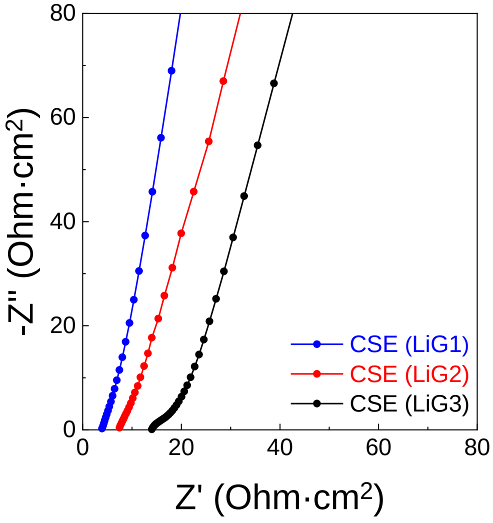

Fig. S17. Nyquist plots of the CSEs (LPSCl/LiGX-containing GPE = 7/3, v/v), in which the composition ratio of LiGX (X = 1, 2, and 3)/ETPTA in the GPE was set to 96.5/3.5 (w/w)

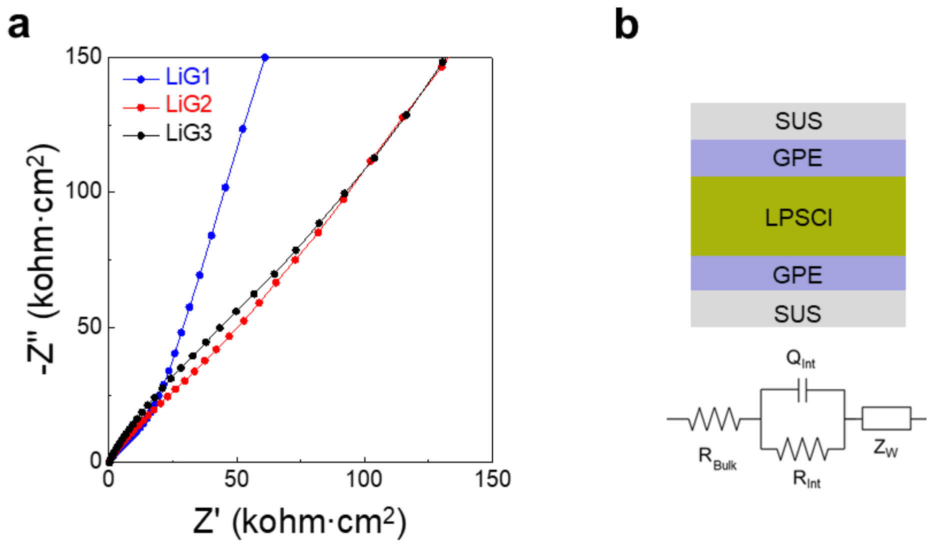

Fig. S18 a Nyquist plots of tri-layered GPE/LPSCl/GPE model electrolytes, in which GPE consisted of LiGX (X = 1, 2, and 3) and ETPTA polymer. **b** Schematic depicting the in-series configuration of the model electrolyte and corresponding equivalent electrical circuit model.

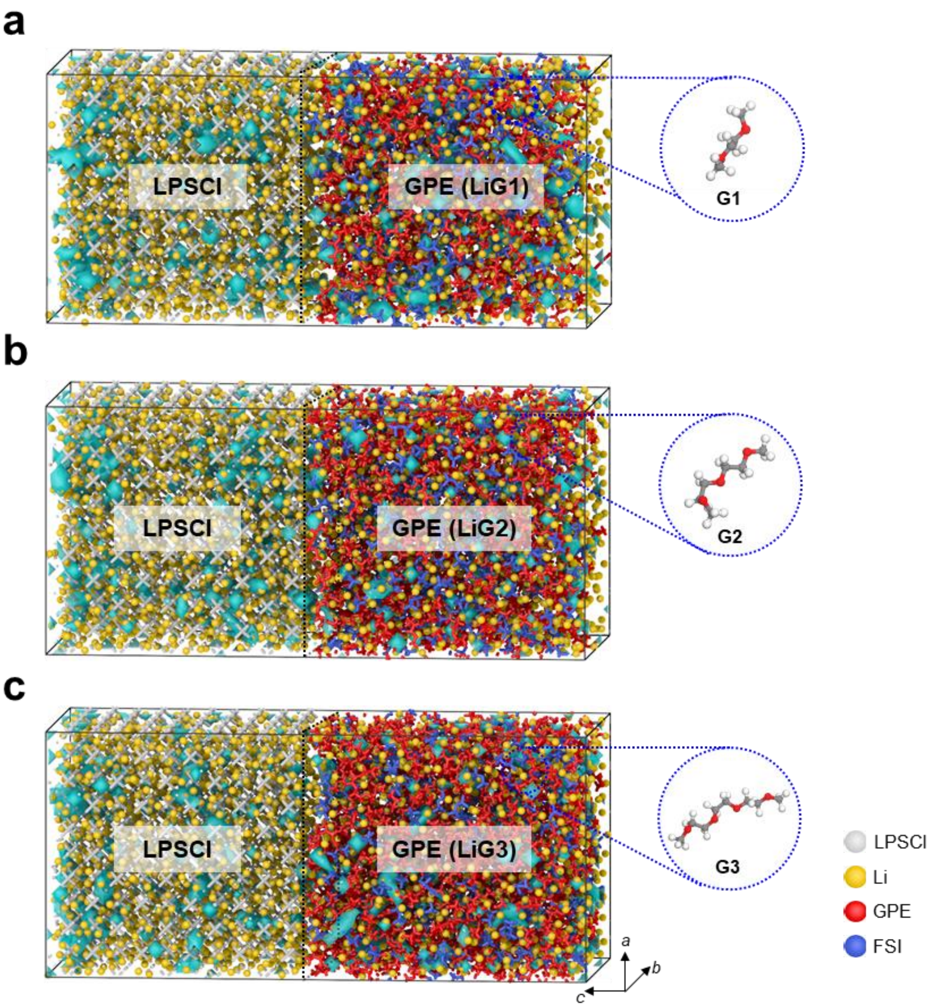

Fig. S19 Model systems used to simulate Li^+ conduction across the LPSCl-GPE interface, in which the GPE contained different Li^+-glyme complexes: **a** LiG1, **b** LiG2, and **c** LiG3. The 3D bluish-green colored polyhedra represent the possible Li^+ diffusion path across the hetero-phase

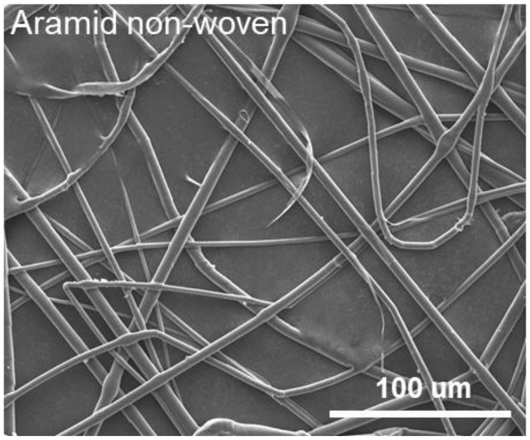

Fig. S20 SEM image of the aramid nonwoven substrate

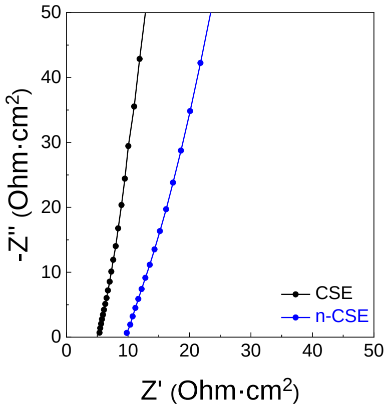

Fig. S21 Nyquist plots of the CSE (LPSCl/GPE-30 (containing the LiG1)) and n-CSE (nonwoven-embedded CSE)

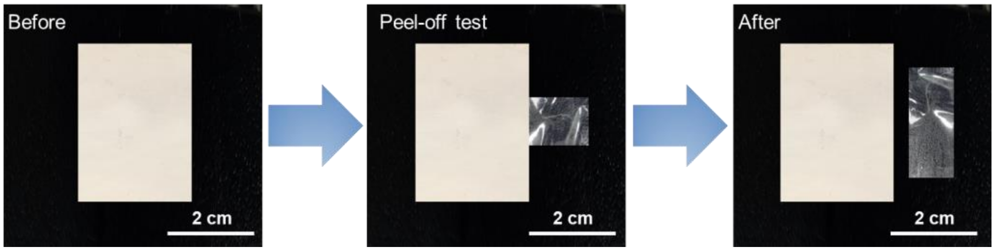

Fig. S22 Photographs of the n-CSE after the peel-off test

a

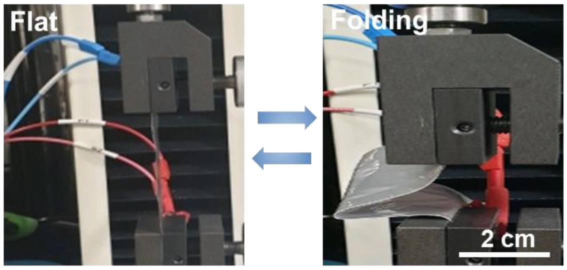

b

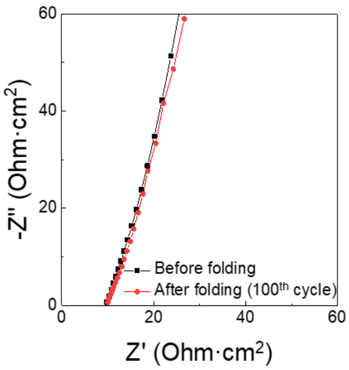

Fig. S23 a Photographs showing the folding of the n-CSE. **b** Nyquist plot of the n-CSE both before and after the 100th folding cycle

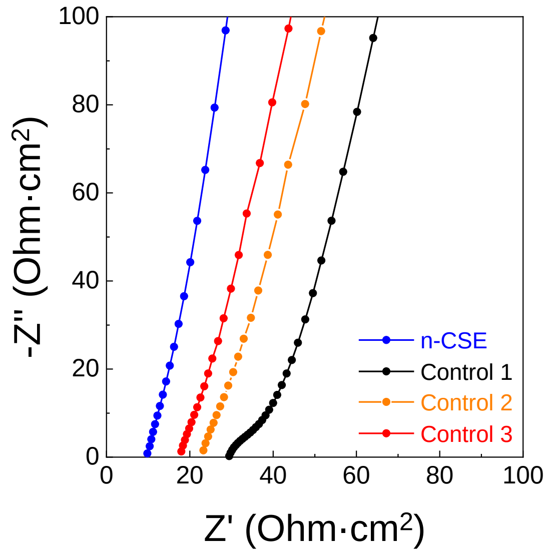

Fig. S24 Nyquist plots of the n-CSE (LPSCl/GPE (LiG1, 3.5wt% ETPTA (30 MPa)), control 1 (LiG3, 220 MPa) consisting of LPSCl and GPE1 (LiG3 and 15 wt.% ETPTA), control 2 (LiG1, 220 MPa) consisting of LPSCl and GPE2 (LiG1 and 15 wt.% ETPTA), and control 3 (LiG3, 30 MPa) consisting of LPSCl and GPE3 (LiG3 and 3.5 wt.% ETPTA)

Table S1 Comparison of the R_{Bulk} (ohm·cm^2) and R_{Int} (kohm·cm^2) values fitted from the EIS profiles of the tri-layered GPE/LPSCl/GPE model electrolyte

	R_{Bulk} (ohm·cm^2)		R_{Int} (kohm·cm^2)
	**LPSCl**	**GPE**	**LPSCl-GPE**
Tri-layer CSE (GPE-LPSCl-GPE)	24.7	44.7	83.6

Table S2 Quantitative analysis of the MAS ^{7}Li NMR spectra of the symmetric cells (^{6}Li|CSE|^{6}Li) both before and after the cycling test (900 h), with a focus on the change in the peak intensity at 1.05 ppm (for the LPSCl) and –1.17 ppm (for the GPE), respectively

	**Before the cycling test**			**After the cycling test**		
	**LPSCl**	**GPE**	**LPSCl/GPE**	**LPSCl**	**GPE**	**LPSCl/GPE**
CSE-30	227.3	23.0	9.9	54.5	10.3	5.3
CSE-220	221.2	32.3	6.9	130.1	19.0	6.8

Table S3 Composition ratios of the model CSEs (tri-layered vs. bi-phasic)

-Volume of the model CSEs (tri-layered and bi-phasic)

Model CSE	CSE Configuration	CSE components	Weight (mg)	Density (g mL^{-1})	Volume (μL)
Tri-layered	LPSCl/GPE-220/LPSCl	LPSCl	140.0	1.86	75.3
		GPE-220	5.4	1.37	4.0
	LPSCl/GPE-30/LPSCl	LPSCl	140.0	1.86	75.3
		GPE-30	5.7	1.42	4.0
Bi-phasic	(LPSCl/GPE-220) × 2	LPSCl	140.0	1.86	75.3
		GPE-220	5.4	1.37	4.0
	(LPSCl/GPE-30) × 2	LPSCl	140.0	1.86	75.3
		GPE-30	5.7	1.42	4.0

- Volume ratio of the model CSEs (tri-layered and bi-phasic)

Model CSE	CSE Configuration	Total volume (μL)	CSE components	Volume (μL)	Volume ratio (vol.%)
Tri-layered	LPSCl/GPE-220/LPSCl	79.3	LPSCl	75.3	95
			GPE-220	4.0	5
	LPSCl/GPE-30/LPSCl	79.3	LPSCl	75.3	95
			GPE-30	4.0	5
Bi-phasic	(LPSCl/GPE-220) × 2	79.3	LPSCl	75.3	95
			GPE-220	4.0	5
	(LPSCl/GPE-30) × 2	79.3	LPSCl	75.3	95
			GPE-30	4.0	5

Volume ratios of the model CSEs (tri-layered and bi-phasic) were obtained using the following Eq. S1:

$$\text{Volume ratio (vol.\%)} = \frac{\text{Volume}_{\text{LPSCl (or GPE)}}}{\text{Total volume (LPSCl + GPE)}} \qquad \text{(S1)}$$

Table S4 Porosity of the bi-phasic CSE with and without the GPE

CSE configuration	CSE components	A_i	M (g)	D (cm)	t (cm)	ρ (g cm^{-3})
(LPSCl/GPE-30) × 2	LPSCl	0.961	0.14	1.3	0.06	1.86
	GPE-30	0.039	0.0057	1.3	0.003	1.42

Sample name	Inclusion of GPE	Porosity (%)
LPSCl-LPSCl (LPSCl × 2)	X	9.1
Bi-phasic CSE ((LPSCl/GPE-30) × 2)	O	5.2

Calculation details on the porosity of the bi-phasic CSE

Porosity value of the bi-phasic CSE was obtained using the following Eq. S2.

$$\text{Porosity [\%]} = 100 - 100\sum(A_i M/\rho_i)/[(\pi/4)\cdot D^2 \cdot t] \qquad \text{(S2)}$$

Where A_i: weight fraction of component i in the bi-phasic CSE; M: weight of the sample (g); D: diameter of the sample (cm); t: thickness of the sample (cm); ρ: apparent density (g cm^{-3})

Table S5 Porosity of the tri-layered CSE with and without the GPE-30

CSE configuration	CSE components	A_i	M (g)	D (cm)	t (cm)	ρ (g cm^{-3})
LPSCl/GPE-30/LPSCl	LPSCl	0.961	0.14	1.3	0.06	1.86
	GPE-30	0.039	0.0057	1.3	0.003	1.42

Sample name	Inclusion of GPE-30	Porosity (%)
LPSCl-LPSCl (LPSCl × 2)	X	9.1
Tri-layered CSE (LPSCl-GPE30-LPSCl)	O	5.2*

* The UV-cured self-standing GPE-30 layer (thickness ~ 30 μm) was placed between the two self-standing LPSCl layers (thickness ~ 300 μm) and followed by pressing at 74 MPa.

Calculation details on the porosity of the tri-layered CSE

Porosity values of the tri-layered CSE was obtained using the following Eq. S3:

$$\text{Porosity [\%]} = 100 - 100\sum(A_i M/\rho_i)/[(\pi/4)\cdot D^2 \cdot t] \qquad \text{(S3)}$$

Where A_i: weight fraction of component i in the tri-layered CSE; M: weight of the sample (g); D: diameter of the sample (cm); t: thickness of the sample (cm); ρ: apparent density (g cm^{-3})

Table S6 Comparison of the R_{Int} (ohm·cm^2) and R_{CT} (ohm·cm^2) values fitted from the EIS profiles of the SSB full cells with the n-CSE (vs. control 1 and control 2) both before and after the cycling test

**Electrolyte**	**1st cycle**		**100st cycle**	
	**R_{Int} (ohm·cm^2)**	**R_{CT} (ohm·cm^2)**	**R_{Int} (ohm·cm^2)**	**R_{CT} (ohm·cm^2)**
n-CSE (LiG1, 30 MPa)	15.6	96.5	21.8	135.1
Control 1 (LiG3, 30 MPa)	27.9	153.2	61.4	337.0
Control 2 (LiG1, 220 MPa)	19.0	130.1	32.3	221.2

Table S7 Calculation details for the volumetric energy densities (excluding the packaging substances) of the SSB full cell with the n-CSE

**C/A (mAh cm^{-2})**	**Nominal voltage (V)**	**$T_{cathode}$ (μm)**	**T_{anode} (μm)**	**$T_{eletrolyte}$ (μm)**	**$T_{current\ collector}$ (μm)**	**T_{total} (μm)**	**Energy density (Wh L^{-1})**
3.5	3.73	100.2	106.8	40.0	25.0	272.0	**480.0**

As shown in Fig. 5i, the volumetric energy density of the SSB full cell is plotted. The equation be derived according to,

$$\text{Volumetric energy density (Wh L}^{-1}) = \frac{\text{Energy}}{\text{Thickness of the cell}} = \frac{\text{Nominal Voltage} \times \text{C/A}}{T_{cathode} + T_{anode} + T_{electrolyte} + T_{current\ collector}}$$

where $T_{cathode}$, T_{anode}, $T_{electrolyte}$, and $T_{current\ collector}$ are the thickness of cathode, anode, electrolyte and current collector (Al (15 μm) and Ti (10 μm)), respectively.

Table S8 Comparison between the SSB bi-cell containing the n-CSE (this study) and previously reported CSE-based SSBs, with a focus on the thickness of solid electrolytes, Li^+ conduction characteristics, cell components, areal mass loading of electrodes, N/P ratios, and volumetric energy densities. Despite the extensive reports on the CSEs, very few studies explicitly provided cell energy densities and N/P ratios. For this reason, the cell energy densities of the previous works were indirectly estimated using the physical/electrochemical results of the electrodes and CSEs.

Electrolyte composition	Electrolyte thickness	Electrolyte Area	Ionic conductivity	Cathode	Mass loading	Anode	n/p	Charge cut-off voltage (V)	Volumetric Energy Density	Refs.
	(μm)	(mm^2)	mS cm^{-1}		(mg cm^{-2})			(V)	Wh L_{cell}^{-1}	
LPSCl/GPE (LiG1/ETPTA)	40	4800	0.41 (25°C)	NCM711	39.0	Graphite	1.1	2.5-4.3	480.0 (at 25 °C)	This work
$77.5Li_2S$-$22.5P_2S_5$/ Methyl-imine	63.7	-	0.092 (25°C)	FeS_2	3.8	Li-In	-	1.0-3.0	100.0	[S1]
LLZO/PEO (LiTFSI)	150	-	0.9 (60°C)	LFP	2.0-3.0	Li	-	3.0-3.8	-	[S2]
LATP/PEGDA-PDMS	1000	-	0.0024 (25°C)	-	-	-	-	-	-	[S3]
LLZTO/PEO-PEG (LiTFSI)	100	-	0.0624 (25°C)	LFP	2.0-3.0	Li	-	2.6-4.0	-	[S4]
LLZO/PEO (LITFSI)	150	-	0.18 (25°C)	-	-	-	-	-	-	[S5]
$Li_{5.4}PS_{4.4}Cl_{1.6}$/PTFE	30	-	8.4 (25°C)	NCM523	11.6	Li	9.2 (*)	2.8-4.2	182.8 (*)	[S6]
Ga-LLZO/PEO	50	-	0.072 (30°C)	LFP	-	Li	-	2.4-3.8	-	[S7]
LGPS/PEO (LiTFSI)	-	-	0.22 (25°C)	-	-	-	-	-	-	[S8]
$70Li_2S$-$30P_2S_5$/Kevlar fiber	100	-	2.4 (25°C)	-	-	-	-	-	-	[S9]
LPSCl/LiG3-NBR	70	-	0.33 (30°C)	NCM711	36.1	Li-In	-	3.0-4.3	407.7 (*)	[S10]

LLZO-Ga/PVDF-HFP (LiFSI)/TEP/FEC	55	-	1.84 (20℃)	NCM532	5.0	Li	-	2.8-4.3	-	[S11]
Al-LLZO/PEO ($LiClO_4$)	-	-	0.009 (-)	-	-	-	-	-	-	[S12]
LLZTO@PDA/PEO (LiTFSI)	-	-	0.11 (30℃)	LFP	1.0	Li	-	3.0-3.9	-	[S13]
LGPS/PEO-Pyr14TFSI (LiTFSI)	-	-	0.54 (-)	NCM811	-	Li-In	-	2.0-3.6	-	[S14]
LLZTO/PEO (LiTFSI)	60	-	0.023 (30℃)	LFP	-	Li	-	2.8-3.8	-	[S15]
NASICON-$LiZr_2(PO_4)_3$/PEO (LiTFSI)	200	-	0.12 (30℃)	NCM811	3.0-5.0	Li	-	2.8-4.3	-	[S16]
LGPS/CTMS/PEG-PEO (LiTTFSI)	48	-	0.983 (25℃)	LFP	-	Li	-	2.5-4.0	-	[S17]
LATP/PEO (LiTFSI)	25	-	0.035 (20℃)	-	-	-	-	-	-	[S18]
LLATO@Li_3PO_4/PVDF-HFP (LiTFSI)/1 M $LiPF_6$ in EC/DMC/DEC	80	-	0.51 (25℃)	LFP	-	Li	-	2.5-4.2	-	[S19]
LLZTO/SN (LiTFSI)/PTFE/Nylon mesh	100	-	0.12 (25℃)	NCM532	3.5	Li-FEC	-	2.5-4.3	-	[S20]
LGPS/PFPE/PVDF-HFP (LiTFSI)	-	-	0.18 (25℃)	LFP	1.5	Li	-	2.5-3.8	-	[S21]
3D LLZAO/PEO (LiTFSI)	240	-	0.251 (25℃)	LFP	1.5	Li	-	2.5-4.2	-	[S22]
LATPO/Cellulose acetate-PEG (LiTFSI)/Pyr_{13}TFSI	45	-	0.132 (60℃)	LFP	2.5	Li	-	2.5-4.0	-	[S23]
LLZO/PEO ($LiClO_4$)	1000	-	0.0088 (25℃)	-	-	-	-	-	-	[S24]
Mxene/PEO (LiTFSI)	-	-	0.022 (28℃)	LFP	2.9	Li	-	2.5-4.0	-	[S25]

LPS/PPTA nonwoven	70	-	-	NCM622	6.4	Graphite	~ 2.4	2.5-4.2	109.0	[S26]
PBA-$LiClO_4$/ $Li_{1.5}Al_{0.5}Ge_{1.5}(PO_4)_3$	75	-	-	NCM622	6	Li	-	3.0-4.2	120.9 (*)	[S27]
$Li_7La_3Zr_2O_{12}$/PEO (LiTFSI)	200	-	-	LCO	11.3	Li	-	3.0-4.2	181.5 (*)	[S28]
β-Li_3PS_4/PEO (LiTFSI)	121	-	-	NCM622	7.6	Li	-	3.0-4.2	146.1 (*)	[S29]
Li_2S_6/PEO (LiTFSI)	200	-	0.17	LFP	3-5	Li	-	2.8-3.8	-	[S30]
PEC/LiMNT/FEC/PTFE (LiFSI)	70	-	0.83 (-)	NCM532	2.0-3.0	3D Li	-	2.5-4.3	-	[S31]

*Estimated from the data provided in the corresponding reference

Supplementary References

[S1] J. M. Whiteley, P. Taynton, W. Zhang and S. H. Lee, Ultra-thin solid-state li-ion electrolyte membrane facilitated by a self-healing polymer matrix. Adv. Mater. **27**, 6922–6927 (2015). https://doi.org/10.1002/adma.201502636

[S2] J. Bae, Y. Li, F. Zhao, X. Zhou, Y. Ding and G. Yu, Designing 3D nanostructured garnet frameworks for enhancing ionic conductivity and flexibility in composite polymer electrolytes for lithium batteries. Energy Storage Mater. **15**, 46–52 (2018). https://doi.org/10.1016/j.ensm.2018.03.016

[S3] X. Liu, S. Peng, S. Gao, Y. Cao, Q. You, L. Zhou, Y. Jin, Z. Liu and J. Liu, Electric-field-directed parallel alignment architecting 3d lithium-ion pathways within solid composite electrolyte. ACS Appl. Mater. Interfaces **10**, 15691−15696 (2018). https://doi.org/10.1021/acsami.8b01631

[S4] L. Chen, Y. Li, S.-P. Li, L.-Z. Fan, C.-W. Nan and J. B. Goodenough, PEO/garnet composite electrolytes for solid-state lithium batteries: From “ceramic-in-polymer” to “polymer-in-ceramic”. Nano Energy **46**, 176–184 (2018). https://doi.org/10.1016/j.nanoen.2017.12.037

[S5] J. Dai, K. Fu, Y. Gong, J. Song, C. Chen, Y. Yao, G. Pastel, L. Zhang, E. Wachsman and L. Hu, Flexible solid-state electrolyte with aligned nanostructures derived from wood. ACS Mater. Lett. **1**, 354−361 (2019). https://doi.org/10.1021/acsmaterialslett.9b00189

[S6] Z. Zhang, L.Wu, D. Zhou, W. Weng and X. Yao, Flexible sulfide electrolyte thin membrane with ultrahigh ionic conductivity for all-solid-state lithium batteries. Nano Lett. **21**, 5233−5239 (2021). https://pubs.acs.org/doi/10.1021/acs.nanolett.1c01344

[S7] Z. Li, H.-M. Huang, J.-K. Zhu, J.-F. Wu, H. Yang, L. Wei, and X. Guo, Ionic conduction in composite polymer electrolytes: case of PEO:Ga-LLZO composites. ACS Appl. Mater. Interfaces **11**, 784−791 (2019). https://doi.org/10.1021/acsami.8b17279

[S8] J. Zheng, P. Wang, H. Liu and Y. Hu, Interface-enabled ion conduction in $Li_{10}GeP_2S_{12}$–poly(ethylene oxide) hybrid electrolytes. ACS Appl. Energy Mater. **2**, 1452−1459 (2019). https://doi.org/10.1021/acsaem.8b02008

[S9] T. Yersak, J.R. Salvador, R.D. Schmidt and M. Cai, Hot Pressed, Fiber-reinforced $(Li_2S)_{70}(P_2S_5)_{30}$ solid-state electrolyte separators for Li metal batteries. ACS Appl. Energy Mater. **2**, 3523−3531 (2019). https://doi.org/10.1021/acsaem.9b00290

[S10] D. Y. Oh, Y. J. Nam, K. H. Park, S. H. Jung, K. T. Kim, A. R. Ha and Y. S. Jung, Slurry-fabricable Li^+-conductive polymeric binders for practical all-solid-state lithium-ion batteries enabled by solvate ionic liquids. Adv. Energy Mater. **9**, 1802927 (2019). https://doi.org/10.1002/aenm.201802927

[S11] D. Xu, J. Su, J. Jin, C. Sun, Y. Ruan, C. Chen and Z. Wen, In situ generated fireproof gel polymer electrolyte with $Li_{6.4}Ga_{0.2}La_3Zr_2O_{12}$ as initiator and ion-conductive filler. Adv. Energy Mater. **9**, 1900611 (2019). https://doi.org/10.1002/aenm.201900611

[S12] W. Zaman, N. Hortance, M. B. Dixit, V.D. Andrade and K.B. Hatzell, In situ generated fireproof gel polymer electrolyte with $Li_{6.4}Ga_{0.2}La_3Zr_2O_{12}$ as initiator and ion-conductive filler. J. Mater. Chem. A **7**, 23914−23921 (2019). https://doi.org/10.1039/C9TA05118J

[S13] Z. Huang, W. Pang, P. Liang, Z. Jin, N. Grundish, Y. Li and C.-A. Wang, A dopamine modified $Li_{6.4}La_3Zr_{1.4}Ta_{0.6}O_{12}$/PEO solid-state electrolyte: enhanced thermal and electrochemical properties. J. Mater. Chem. A. **7**, 16425−16436 (2019). https://doi.org/10.1039/C9TA03395E

[S14] W. Cho, J. Park, K. Kim, J. Yu and G. Jeong, Sulfide-compatible conductive and adhesive glue-like interphase engineering for sheet-type all-solid-state battery. Small **17**, 1902138 (2019). https://doi.org/10.1002/smll.201902138

[S15] H. Huo, Y. Chen, X. Yang, X. Guo, and X. Sun, Rational design of hierarchical “ceramic-in-polymer” and “polymer-in-ceramic” electrolytes for dendrite-free solid-state batteries. Adv. Energy Mater. **9**, 1804004 (2019). https://doi.org/10.1002/aenm.201804004

[S16] N. Wu, P.-H. Chien, Y. Li, A. Dolocan, H. Xu, B. Xu, N. S. Grundish, H. Jin, Y.-Y. Hu, and J. B. Goodenough, Fast Li^+ conduction mechanism and interfacial chemistry of a NASICON/polymer composite electrolyte. J. Am. Chem. Soc. **142**, 2497–2505 (2020). https://doi.org/10.1021/jacs.9b12233

[S17] K. Pan, L. Zhang, W. Qian, X. Wu, K. Dong, H. Zhang and S. Zhang, A flexible ceramic/polymer hybrid solid electrolyte for solid-state lithium metal batteries. Adv. Mater. **32**, 2000399 (2019). https://doi.org/10.1002/adma.202000399

[S18] M. J. Palmer, S. Kalnaus, M. B. Dixit, A. S. Westover, K. B. Hatzell, N. J. Dudney and X. C. Chen, A three-dimensional interconnected polymer/ceramic composite as a thin film solid electrolyte. Energy Storage Mater. **26**, 242–249 (2020). https://doi.org/10.1016/j.ensm.2019.12.031

[S19] M. J. Palmer, J. Bright, B. Chen, P. Chen, P. Zheng, X. Gao, B. Liu, S. Kasani, X. Zhang and N. Wu, Chemical interaction and enhanced interfacial ion transport in a ceramic nanofiber–polymer composite electrolyte for all-solid-state lithium metal batteries. J. Mater. Chem. A **8**, 7261–7272 (2020). https://doi.org/10.1039/C9TA12495K

[S20] T. Jiang, P. He, G. Wang, Y. Shen, C.-W. Nan and L.-Z. Fan, Solvent-free synthesis of thin, flexible, nonflammable garnet-based composite solid electrolyte for all-solid-state lithium batteries. Adv. Energy Mater. **10**, 1903376 (2020). https://doi.org/10.1039/C9TA12495K

[S21] L. Cong, Y. Li, J. Jie, Y. Liu, L. Sun and H. Xie, Unlocking the Poly (vinylidene fluoride-co-hexafluoropropylene)/$Li_{10}GeP_2S_{12}$ composite solid-state electrolytes for dendrite-free Li metal batteries assisting with perfluoropolyethers as bifunctional adjuvant. J. Power Sources **446**, 227365 (2020). https://doi.org/10.1016/j.jpowsour.2019.227365

[S22] D. Cai, D. Wang, Y. Chen, S. Zhang, X. Wang, X. Xia and J. Tu, A highly ion-conductive three-dimensional LLZAO-PEO/LiTFSI solid electrolyte for high-performance solid-

state batteries. Chem. Eng. Sci. **394**, 124993 (2020). https://doi.org/10.1016/j.cej.2020.124993

[S23] Q. Ma, X.-X. Zeng, J. Yue, Y.-X. Yin, T.-T. Zou, J.-Y. Liang, Q. Deng, X.-W. Wu and Y.-G. Guo, Viscoelastic and nonflammable interface design–enabled dendrite-free and safe solid lithium metal batteries. Adv. Energy Mater. **9**, 1803854 (2019). https://doi.org/10.1002/aenm.201803854

[S24] M. B. Dixit, W. Zaman, Y. Bootwala, Y. Zheng, M. C. Hatzell and K. B. Hatzell, Scalable manufacturing of hybrid solid electrolytes with interface control. ACS Appl. Mater. Interfaces, **11**, 45087−45097 (2019). https://doi.org/10.1021/acsami.9b15463

[S25] Q. Pan, Y. Zheng, S. Kota, W. Huang, S. Wang, H. Qi, S. Kim, Y. Tu, M. W. Barsoum and C. Y. Li, 2D MXene-containing polymer electrolytes for all-solid-state lithium metal batteries. Nanoscale Adv. **1**, 395–402 (2019). https://doi.org/10.1039/C8NA00206A

[S26] D. Y. Oh, D. H. Kim, S. H. Jung, J.-G. Han, N.-S. Choi and Y. S. Jung, Single-step wet-chemical fabrication of sheet-type electrodes from solid-electrolyte precursors for all-solid-state lithium-ion batteries. J. Mater. Chem. A **5**, 20771−20779 (2017). https://doi.org/10.1039/C7TA06873E

[S27] M. S. Park, Y. C. Jung, and D. W. Kim, Hybrid solid electrolytes composed of poly(1,4-butylene adipate) and lithium aluminum germanium phosphate for all-solid-state $Li/LiNi_{0.6}Co_{0.2}Mn_{0.2}O_2$ cells. Solid State Ion. **315**, 65–70 (2018). https://doi.org/10.1016/j.ssi.2017.12.007

[S28] H. Wakayama, H. Yonekura and Y. Kawai, Three-dimensional bicontinuous nanocomposite from a self-assembled block copolymer for a high-capacity all-solid-state lithium battery cathode. Chem. Mater. **28**, 4453–4459 (2016). https://doi.org/10.1021/acs.chemmater.6b01665

[S29] T. Ates, M. Keller, J. Kulisch, T. Adermann and S. Passerini, Development of an all-solid-state lithium battery by slurry-coating procedures using a sulfidic electrolyte. Energy Storage Mater. **18**, 31261–31264 (2018). https://doi.org/10.1016/j.ensm.2018.11.011

[S30] R. Fang, B. Xu, N. S. Grundish, Y. Xia, Y. Li, C. Lu, Y. Liu, N. Wu and J. B. Goodenough, Li_2S_6-integrated PEO-based polymer electrolytes for all-solid-state lithium-metal batteries. Angew. Chem. **133**, 17842–17847 (2021). https://doi.org/10.1002/ange.202106039

[S31] L. Chen, W. Li, L. Z. Fan, C. W. Nan and Q. Zhang, Intercalated electrolyte with high transference number for dendrite-free solid-state lithium batteries. Adv. Funct. Mater. **29**, 1901047 (2019). https://doi.org/10.1002/adfm.201901047
